# Efficacy of a Remote Person-Centered Intervention Using an eHealth Platform and Telephone Support for Persons With Chronic Pain: Randomized Controlled Trial

**DOI:** 10.2196/91887

**Published:** 2026-08-03

**Authors:** Åse Lundin, Veronica Lilja, Inger Ekman, Paulin Andréll, Mari Lundberg, Markus Saarijärvi, Vivi-Anne Segertoft, Mahboubeh Goudarzi, Andreas Fors, Sara Wallström

**Affiliations:** 1 Institute of Health and Care Sciences, Sahlgrenska Academy, University of Gothenburg, Arvid Wallgrens backe, hus 1, Gothenburg, 405 30, Sweden, 46 31-786 00 0; 2 University of Gothenburg Centre for Person-Centred Care (GPCC), Sahlgrenska Academy, University of Gothenburg, Gothenburg, Sweden; 3 Department of Medicine, Geriatrics and Emergency Care, Sahlgrenska University Hospital/Östra, Gothenburg, Sweden; 4 Department of Anaesthesiology, Intensive Care Medicine and Pain Medicine, Sahlgrenska University Hospital, Gothenburg, Region Västra Götaland, Sweden; 5 Department of Anaesthesiology and Intensive Care Medicine, Institute of Clinical Sciences at Sahlgrenska Academy, University of Gothenburg, Gothenburg, Sweden; 6 Department of Health Promoting Science, Sophiahemmet University, Stockholm, Sweden; 7 Pain in Motion Research Group (PAIN), Department of Physiotherapy, Human Physiology and Anatomy, Faculty of Physical Education & Physiotherapy, Vrije Universiteit Brussel, Brussels, Belgium; 8 Department of Cardiology, Danderyd Hospital, Stockholm, Sweden; 9 Department of Neurobiology, Care Sciences and Society, Karolinska Institute, Stockholm, Sweden; 10 Research, Education, Development and Innovation, Primary Health Care, Gothenburg, Region Västra Götaland, Sweden; 11 Department of Forensic Psychiatry, Sahlgrenska University Hospital, Gothenburg, Region Västra Götaland, Sweden; 12 Centre for Ethics, Law and Mental Health (CELAM), University of Gothenburg, Gothenburg, Sweden

**Keywords:** chronic pain, person-centered care, person-centered, sick leave, self-efficacy, randomized controlled trial, eHealth, telehealth

## Abstract

**Background:**

Chronic pain is a growing global public health challenge and the leading cause of years lived with disability. It significantly impacts various aspects of daily life, including participation in working life, resulting in increased sick leave and substantial burdens at both the personal and societal levels. Person-centered care (PCC) is a practiced ethic that recognizes the patient as a partner in care. Partnership is established through incorporating the person’s narrative, shared decision-making, and documentation of jointly agreed goals. Remotely delivered PCC interventions have been shown in other studies to improve self-efficacy and facilitate return to work among persons on sick leave. However, little is known about how self-efficacy and sick leave are affected when a PCC intervention is delivered remotely to persons with chronic pain.

**Objective:**

This study aimed to evaluate the efficacy of a home-based person-centered intervention consisting of telephone support and an eHealth platform among persons on sick leave due to chronic pain.

**Methods:**

A 2-arm, nonblinded randomized controlled trial was conducted. Participants aged 18 to 65 years on sick leave due to chronic, nonmalignant pain lasting more than 3 months were recruited from 10 primary health care centers in Gothenburg, Sweden. Participants were randomly allocated 1:1 to either the control group or the intervention group. Both groups received usual care; the intervention group additionally participated in a 6-month PCC intervention via telephone and an eHealth platform. The primary outcome was a composite score consisting of change in general self-efficacy and sick leave at the 6-month follow-up. Self-efficacy was assessed using the Swedish version of the 10-item General Self-Efficacy Scale, and sick leave was assessed based on participants’ self-reported percentage of sick leave in relation to full-time work. The primary outcome was analyzed according to the intention-to-treat principle using the Mantel-Haenszel chi-square trend test.

**Results:**

A total of 654 patients were assessed for eligibility, of whom 59 were included in the final analysis: 29 in the intervention group and 30 in the control group. More participants in the control group (11/30, 36.7%) than in the intervention group (3/29, 10.3%) showed deterioration, resulting in a significant difference in the composite score between the groups at the 6-month follow-up (*P*=.04), favoring the intervention. This significance also remained in the nonimputed analysis (*P*=.04).

**Conclusions:**

This study suggests that PCC via telephone and an eHealth platform may influence the level of sick leave and self-efficacy among persons with chronic pain. Since the control group deteriorated while the intervention group largely remained unchanged, PCC may play a protective role in supporting persons with chronic pain in returning to work. Further studies are warranted to confirm these findings.

## Introduction

Chronic pain affects 19% to 20% of the population in Europe and North America [1,2] and is the single greatest cause of years lived with disability [3]. Chronic pain is associated with psychiatric comorbidity, cardiovascular diseases, and cancer [4], an elevated risk of suicidal ideation and suicide attempts [5], and premature mortality [6]. Furthermore, chronic pain negatively impacts quality of life and daily activities and has repercussions for the person’s social and family environment [7]. Chronic pain significantly impacts the ability to work [8,9], leading to increased sick leave and economic burdens at both the personal and societal levels [10]. Work absences account for the majority of the estimated cost of chronic pain and, in European countries, reach 4% to 10% of gross domestic product [11,12]. In 2021, the economic burden of chronic pain in the United States was estimated at US $722.8 billion [13].

Guidelines for best practices in chronic pain management advocate a biopsychosocial and multidisciplinary approach, including physical therapy and exercise, pharmacological treatment, and psychological therapies [14]. In Sweden, the majority of patients with chronic pain are treated within the primary health care system [15]. Despite existing recommendations and guidelines, challenges remain in the provision of chronic pain management in primary health care settings, where an inventory has highlighted deficiencies and unjustified variations in care and treatment [15]. In the European Pain Federation 2025 research strategy, 5 priority themes for pain research were identified as deserving increased focus and funding. One of these themes concerns the evaluation of new or repurposed treatments, including person-centered approaches [16].

Person-centered care (PCC) is a practiced ethic [17] founded on the establishment of a partnership between the patient and health care professionals (HCPs), and possibly others, such as family members [18,19]. This partnership is based on mutual trust, understanding, and shared knowledge, and is accomplished through genuine engagement, empathetic presence, shared decision-making, and integration of the patient’s beliefs, values, needs, and resources [19,20]. Previous PCC interventions have evaluated remote delivery of an intervention via an eHealth platform developed at the University of Gothenburg through a collaboration among HCPs, information technology developers, researchers, and research partners. The platform’s application and content can be tailored to different target populations across studies. Combining the use of the platform with telephone support, randomized controlled trials (RCTs) have shown improvement in self-reported general self-efficacy (GSE) in persons with chronic heart failure and common mental disorders [21,22]. “Self-efficacy” refers to a person’s belief in their ability to succeed in certain situations [23], and its importance in facilitating return to work has been highlighted in several studies [24-26]. Self-efficacy is important in relation to chronic pain, as higher self-efficacy is associated with positive treatment outcomes for physical functioning and level of disability [27], work status, and lower pain intensity [28], while lower or reduced self-efficacy is a negative predictor of return to work [29].

However, although the telephone support and an eHealth platform have been evaluated in other target groups, little is known about how self-efficacy and sick leave are affected when this type of combined PCC intervention is delivered remotely to persons with chronic pain. Given the substantial impact of chronic pain on personal life, workforce participation, economic stability, and health care systems, addressing chronic pain–related sick leave represents a critical public health priority. Therefore, this study aims to evaluate the efficacy of a person-centered intervention at home, consisting of combined telephone support and an eHealth platform, among persons on sick leave due to chronic pain.

## Methods

### Reporting Guidelines

This study is reported in accordance with the CONSORT-EHEALTH (Consolidated Standards of Reporting Trials of Electronic and Mobile Health Applications and Online Telehealth) checklist (version 1.6.1) (Checklist 1) [30] and the GRIPP2-SF (Guidance for Reporting Involvement of Patients and the Public-Short Form) checklist (Checklist 2) [31].

### Study Design

The EAPER-P (Early Accessible Person-Centered Rehabilitation for Patients With Chronic Pain) trial was a 2-arm, nonblinded, open RCT hypothesizing that, among persons on sick leave due to chronic pain, those who receive a PCC intervention will have an improved composite score combining self-reported self-efficacy and sick leave compared with controls. Accordingly, the null hypothesis assumed that there would be no differences between the intervention and control groups at the end point.

Participants were individually allocated in a 1:1 ratio to the control group or the intervention group. Both groups received usual care (described below) for 6 months; the intervention group additionally participated in a remote PCC intervention consisting of telephone calls with HCPs and an eHealth platform. Because the inclusion rate was slower than expected, the research group deemed it infeasible to continue recruitment until the desired statistical power was achieved. Thus, inclusion was terminated after 22 months in June 2023. No interim analyses were planned a priori or conducted during the course of the RCT. All analyses were performed after study completion.

### Participants and Recruitment

Participants were recruited from 10 primary health care centers in a socioeconomically diverse area of Gothenburg, Sweden. Inclusion criteria were men and women aged 18 to 65 years on sick leave due to chronic (lasting >3 mo), nonmalignant pain with any of the following diagnoses according to the *International Statistical Classification of Diseases and Related Health Problems, 10th Revision* (*ICD-10*) [32]: M25—other joint disorders not elsewhere classified; M54—dorsalgia; M79—other soft tissue disorders not elsewhere classified; or R52—pain not elsewhere classified. Exclusion criteria were being on full-time sick leave for ≥24 months; severe cognitive or physical impairment (ie, preventing the person from using the eHealth support); severe disease other than chronic pain, with expected survival ≤12 months; a documented diagnosis of alcohol or drug abuse; participating in another conflicting RCT; not understanding written and spoken Swedish; not having access to a digital device with internet access; and having no registered address.

### Enrollment and Randomization

Recruitment took place between August 2021 and June 2023. Medical records from the participating health care centers were continuously screened for eligible participants by a designated HCP. Letters containing information about the study, contact details, and a note indicating that the person would be contacted by telephone for further information about the study were sent to eligible participants. Further information about the study was provided over the phone, and those interested in participating were sent written informed consent forms via post together with a prepaid return envelope. After returning a signed consent form, participants were randomized to either the intervention or the control group by the same designated HCP. Randomization was performed using web-based randomization software; dSharp Randomization (dSharp Consulting & Statistiska Konsultgruppen), with stratification by age and sex. Participants were informed about their randomized allocation by telephone.

### Control Group

The control group received usual care. In this study, usual care comprised the standard management of chronic pain provided within the Swedish health care system, with treatment tailored to individual needs and available resources. According to the Swedish national guidelines, management of chronic pain should follow a biopsychosocial approach and may include both pharmacological and nonpharmacological treatments. Care is typically initiated in primary health care, with referral to specialist care when needed [33,34]. Management may be unimodal, consisting of a single intervention, or multimodal, involving coordinated multiprofessional interventions. Common components of usual care include physical exercise, physiotherapeutic interventions, pain education, pharmacological interventions, and psychological interventions, delivered alone or in combination. In this study, the content of usual care was not standardized or controlled by the study and could therefore vary among participants depending on personal needs, health care providers’ decisions, and available treatment options.

### Intervention Group

#### PCC Intervention

The intervention group, in addition to usual care, participated in a PCC intervention consisting of 2 parts: telephone calls with HCPs combined with access to an eHealth platform during a 6-month period. The HCPs carrying out the intervention were 3 registered nurses and a registered physiotherapist. One had extensive previous experience in providing PCC through telephone and eHealth platforms, while the others received initial training prior to the study, consisting of coursework in PCC philosophy equivalent to 7.5 European Credit Transfer and Accumulation System. Thereafter, all HCPs providing the intervention continued training as a group through recurring educational seminars and mentoring sessions on practicing PCC. These were held approximately once a month throughout the intervention and were led by senior researchers experienced in PCC. The HCPs carrying out the intervention operated from a research unit at the hospital and were not involved in the participants’ usual care or connected to the primary health care centers from which the participants were recruited.

To ensure a partnership in PCC, the intervention followed the 3 routines (initiating, working, and safeguarding the partnership) described by Ekman et al [19]. Initiating the partnership means encouraging and actively listening to the participant’s narrative about their pain, life situation, needs, resources, and preferences. Working the partnership involves sharing information, shared deliberation, and decision-making to reach a common understanding and common goals. Safeguarding the partnership includes documenting the patient’s narrative, the mutually agreed health plan, and actions to achieve the shared short-term and long-term goals.

##### Telephone Calls

Participants booked at least 1 initial telephone call with the HCP and then scheduled follow-up calls according to individual needs during the 6-month intervention period. During the calls, the participants were encouraged by the HCPs to narrate their experiences. The HCPs asked open-ended questions about the participants’ current situation, listened, and asked follow-up questions. During the conversations, the participants’ resources were identified, both long- and short-term goals were formulated, and a jointly agreed health plan was created. The health plan contained information relevant to the participant, such as a summary of the participant’s situation, resources and goals, and the support needed to achieve those goals. The health plan was continuously updated and followed up throughout the intervention, usually in connection with a scheduled call. Toward the end of each call, subsequent calls were jointly scheduled with the participant based on their wishes and needs, typically at intervals of 2 to 4 weeks, and documented in the health plan.

##### eHealth Platform

**Figure 1.**
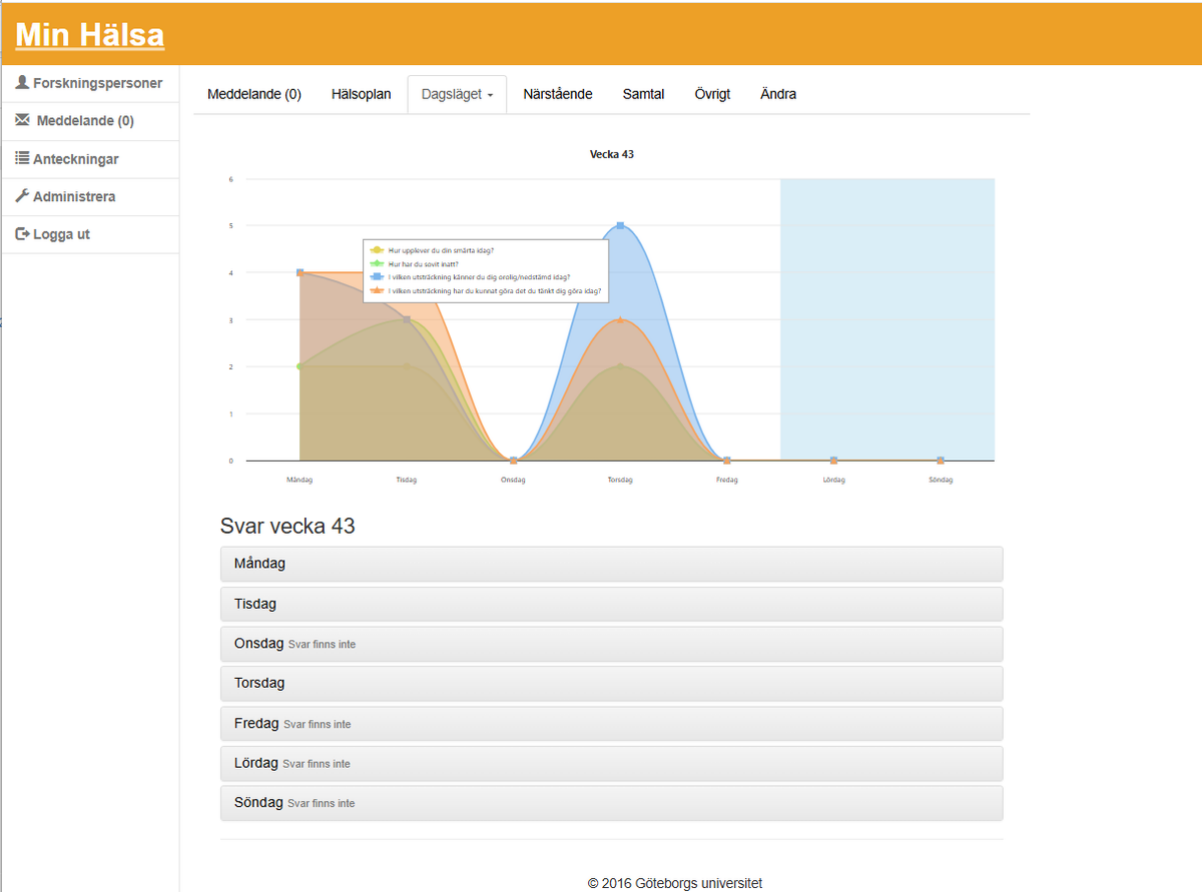
Screenshot of the MyHealth eHealth platform.

The health plan was written down by the HCP or the participant, based on the participant’s preferences, and uploaded to the eHealth platform, MyHealth (Figure 1). Using a personal login on a computer or smartphone, participants were able to access, add to, or revise the health plan at any time on the eHealth platform. They could use the platform to send messages to the HCPs; see the date and time of their next agreed-upon telephone call; invite others (their social networks and/or regular health care contacts) to the platform; and rate their sleep, activities, pain, and anxiety on a 5-point Likert scale. Answers to the questions created a graph that enabled both participants and the HCPs to follow trends. These ratings on the eHealth platform were used to facilitate and tailor conversations during calls with the HCPs; they were not extracted or used in the final statistical analyses.

### Data Collection

At inclusion, data on sex, age, diagnosis, comorbidities, and medications were collected from the medical records by a designated HCP. Participants answered questionnaires by post at baseline and at 3 and 6 months after inclusion (end point). At baseline, self-reported data on country of birth, level of education, occupation, and living situation were collected, while primary outcome data on GSE and sick leave ratio were collected on all 3 occasions. Additionally, self-reported pain intensity was collected on all 3 occasions. This was assessed using an 11-point Numeric Rating Scale, ranging from 0 (no pain) to 10 (worst pain imaginable) [35]. Participants reported their average pain intensity during the preceding week both at rest and during movement. The number of telephone calls and updates to health plans was registered prospectively in study logs throughout the intervention.

Self-efficacy was assessed using the Swedish version [36] of the GSE scale [37]. The GSE scale is a 10-item questionnaire used to assess a person’s belief in their ability to handle problems and associated difficulties [36]. The items in the GSE scale consist of statements answered on a 4-point Likert scale, where 1=“not at all true,” 2=“hardly true,” 3=“moderately true,” and 4=“exactly true.” The total score ranges from 10 to 40, with higher scores corresponding to higher self-efficacy [37]. The scale has shown high internal consistency (Cronbach α=0.91) when examined in relation to work capacity and sick leave in a randomized general population [36]. The GSE has been used as a primary outcome measure in previous evaluations of PCC delivered remotely, aimed toward persons with chronic heart failure and common mental disorders [21,22].

Sick leave was measured based on the participants’ self-reported current percentage of sick leave in relation to full-time work, on a scale of 0‐100. Participants were instructed to report their current level of continuous sick leave rather than isolated sick days due to minor illnesses (eg, colds). According to the Swedish sickness benefit system, which is regulated by national legislation, sick leave is granted and reported at 25%, 50%, 75%, or 100% of regular working hours. Participants reported their level of sick leave using these legally defined categories. Return to work, either part-time or full-time, during the intervention period did not constitute withdrawal from the trial, and participants continued the intervention and follow-up assessments regardless of work status.

### Primary Outcome

The primary outcome consisted of a composite of changes in self-reported GSE scores and sick leave level at the end point (6-mo follow-up). A composite score merges 2 or more relevant variables into a single measure [38]. Initially developed within efficacy trials in cardiology [39], it is now a commonly applied approach in health research and serves as a valuable tool for the evaluation of clinical trials, as it provides statistical advantages, including reduced sample size requirements and shorter follow-up periods [40]. The composite score used in this RCT was defined a priori, and, to provide transparency in reporting, the results of each individual variable were presented separately [40].

Participants were classified as improved, deteriorated, or unchanged. Participants were classified as improved if their GSE score had increased by ≥5 points and if they had a reduced sick leave level compared with baseline. They were classified as deteriorated if their GSE score had decreased by ≥5 points and/or they had increased sick leave compared with baseline. Participants who did not fulfill the criteria for either improvement or deterioration were classified as unchanged. Previous research has considered a 5-point change in GSE to be the threshold for a minimal important change [41,42]. Sick leave level and GSE as a composite score have previously been used in another RCT evaluating PCC [22].

### Power Calculation

Assuming an improvement in the composite score of 20% in the control group and 40% in the intervention group, an α level of .05, 80% power, and a 2-sided Fisher exact test, 91 participants per group were calculated to be needed for inclusion in the study.

### Statistical Analysis

Background characteristics were calculated using descriptive and inferential statistics. Fisher exact test was used for dichotomous variables, the Mann-Whitney *U* test for continuous variables, and the chi-square test for nonordered categorical variables. The primary outcome was analyzed using the Mantel-Haenszel chi-square trend test. Mixed models for repeated measures with an unstructured covariance pattern were used for the outcome of change from baseline to follow-up and were adjusted for the baseline value of the outcome as a covariate in the model. For sick leave, the data were not normally distributed; therefore, robust sandwich estimators were used to ensure valid inference. Least squares means are the model-estimated marginal means for each group at the mean baseline value of the outcome (GSE or sick leave).

As there were no major protocol violations, only an intention-to-treat analysis was performed. Missing outcome data for the composite score (GSE and sick leave level) at the 3- and 6-month follow-ups were imputed using the last observation carried forward. The significance level was set at *P*<.05 (2-sided), and 95% CIs were reported. The statistical analysis plan is available as supplementary material in Multimedia Appendix 1. SAS statistical software (version 9.4; SAS Institute Inc) and IBM SPSS Statistics (version 29; IBM Corp) were used for the calculations.

### Patient and Public Involvement

In this trial, we engaged in patient and public involvement, primarily through working with 2 persons we refer to as research partners. Research partners were patient representatives with extensive experience of chronic pain and sick leave, bringing first-hand perspectives and knowledge regarding the lived experience of the condition, navigating health care systems, and providing perspectives that aided in the understanding of the intervention and the trial results. One of the research partners is a coauthor of this manuscript (VAS).

Additionally, several other research partners have been actively involved in the design phase of the projects preceding our trial, during which the eHealth platform used in our intervention was developed. The eHealth platform was developed at the University of Gothenburg through collaboration among information technology developers, HCPs, researchers, and research partners. The research partners were actively involved in its development through a participatory process, offering insights into its viability and later modifying the platform content to accommodate different target populations [43].

### Ethical Considerations

The study adhered to the Declaration of Helsinki [44]. It was approved by the Swedish Ethical Review Authority (register number: 2020‐02491; amendments 2020‐06936 and 2021‐02753). Written and verbal informed consent was obtained from each participant before participation in the study. Participant confidentiality was ensured by pseudonymization as well as by storing collected data and identifiers with restricted access. No compensation was provided for participation in this study.

## Results

**Figure 2.**
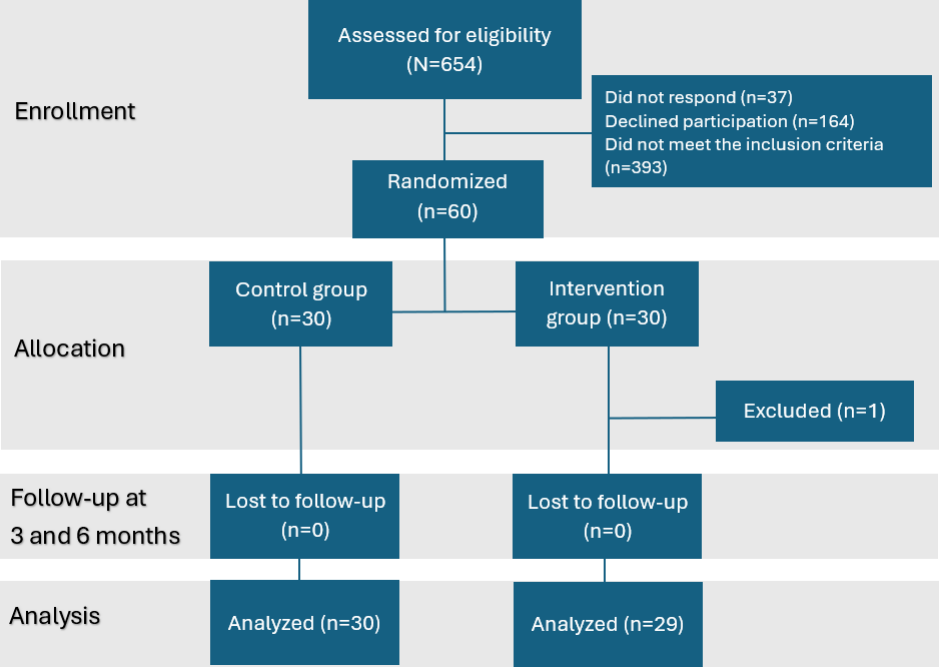
CONSORT (Consolidated Standards of Reporting Trials) flow diagram of the participant inclusion process. Three participants were screened twice but are reported only once in the flowchart.

A total of 654 patients were assessed for eligibility, of whom 393 did not meet the inclusion criteria, 164 declined participation, and 37 did not respond. Therefore, 60 participants were randomized to the control or intervention group. However, after allocation to the groups, 1 person was found to have been erroneously randomized. After exclusion of that person, a total of 59 participants were included in the analyses (intervention group: n=29, 49%; control group: n=30, 51%). A flowchart of enrollment, allocation, follow-up, and analysis is presented in Figure 2. There were no withdrawals, major protocol violations, adverse events, or deaths in either group.

The participants’ mean age was 48.3 (SD 10.1) years, and the majority of participants were women (38/59, 64.4%). There were no statistically significant differences in demographic characteristics between the groups at baseline (Table 1). See the supplementary materials for prespecified medical history (Table S1 in Multimedia Appendix 2) and concomitant medications (Table S2 in Multimedia Appendix 2).

**Table 1.** Demographic and baseline characteristics of the intervention and control groups.

Variable	Intervention group (n=29)	Control group (n=30)
Age (y), mean (SD)	48.4 (10.8)	48.2 (9.7)
Sex, n (%)		
Male	10 (34.5)	11 (36.7)
Female	19 (65.5)	19 (63.3)
Diagnosis (*ICD-10*^a^), n (%)		
M25 (other joint disorders not elsewhere classified)	5 (17.2)	4 (13.3)
M54 (dorsalgia)	12 (41.4)	14 (46.7)
M79 (other soft tissue disorders not elsewhere classified)	7 (24.1)	8 (26.7)
R52 (pain not elsewhere classified)	5 (17.2)	4 (13.3)
Living situation, n (%)		
Married/living together	20 (69.0)	19 (63.3)
Living apart	0 (0.0)	1 (3.3)
Single	9 (31.0)	10 (33.3)
Education, n (%)		
Compulsory school or lower	3 (10.3)	1 (3.3)
Upper secondary school or equivalent	13 (44.8)	15 (50)
Postsecondary vocational school	6 (20.7)	3 (10)
College/university	7 (24.1)	11 (36.7)
Country of birth, n (%)		
Sweden	21 (72.4)	18 (60)
Other	8 (27.6)	12 (40)
Pain intensity when in movement (NRS^b^ score 0-10)		
Mean (SD)	5.90 (1.99)	6.34 (2.53)
Median (range)	7 (2–9)	7 (0‐10)
Pain intensity when resting (NRS score 0-10)		
Mean (SD)	4.48 (2.40)	4.62 (2.81)
Median (range)	4 (0‐9)	5 (0‐10)

a *ICD-10*: *International Statistical Classification of Diseases and Related Health Problems, 10th Revision*.

b NRS: Numeric Rating Scale, where 0=no pain, 10=worst pain imaginable.

The median number of telephone calls in the intervention group during the 6 months was 7 (IQR 6-8; range 2-10). All participants had a documented health plan on the platform, which was updated 1 to 7 times per participant (most often in connection with a telephone call).

**Figure 3.**
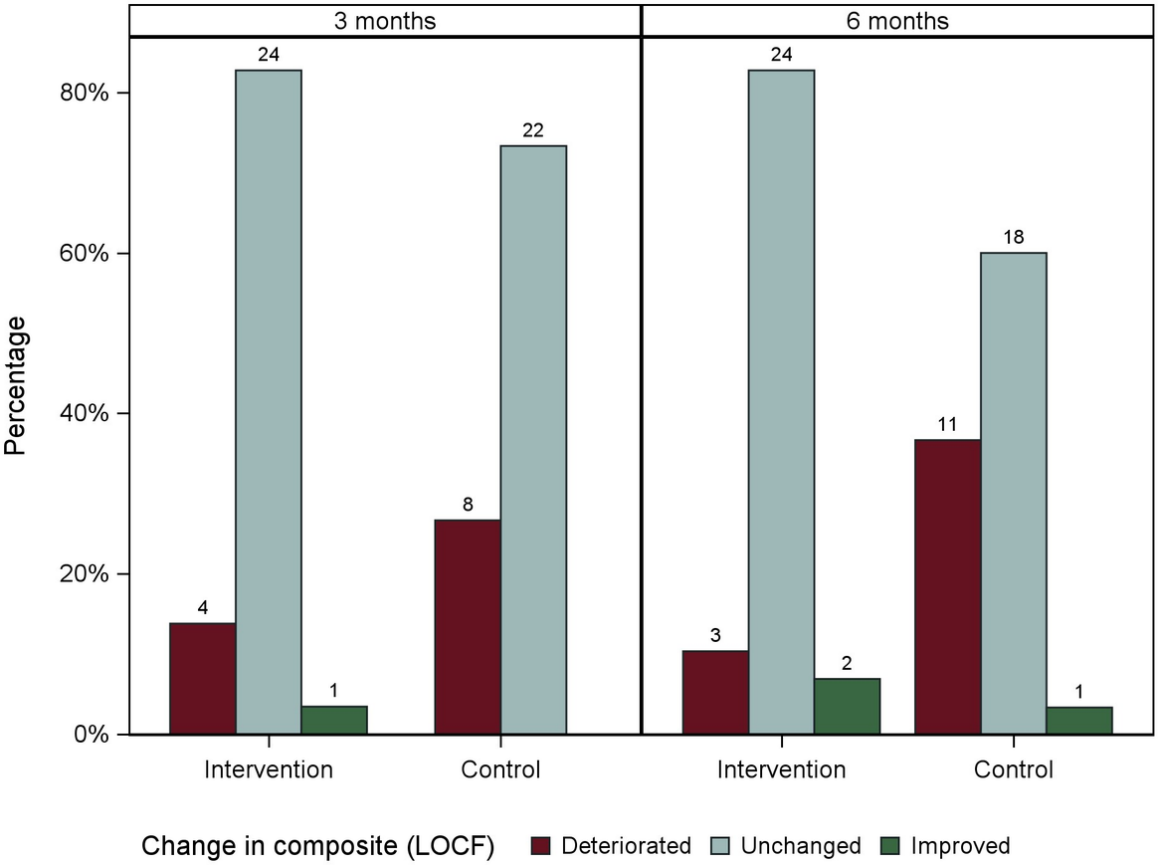
Change from the baseline composite (general self-efficacy and sick leave) score at the 3- and 6-month follow-ups. Missing data were imputed using the last observation carried forward (LOCF).

At 6 months, there was a statistically significant difference (*P*=.04) in the primary outcome between the control group and the intervention group (Table 2). More participants in the control group (11/30, 36.7%) than in the intervention group (3/29, 10.3%) showed deterioration at 6 months (Figure 3). Additionally, more participants in the intervention group (24/29, 82.8%) compared with the control group (18/30, 60.0%) remained unchanged at the 6-month follow-up (Figure 3). A significant difference in the composite score was also present in the nonimputed analysis (*P*=.04; Table S3 in Multimedia Appendix 3).

**Table 2.** Composite scores (sick leave and general self-efficacy) at 3 and 6 months^a^.

Outcome	3-month follow-up				6-month follow-up			
	Intervention (n=29), n (%)	Control (n=30), n (%)	Difference in proportion (95% CI)	*P* value	Intervention (n=29), n (%)	Control (n=30), n (%)	Difference in proportion (95% CI)	*P* value
Composite score				.23				.04
Deteriorated	4 (13.8)	8 (26.7)	−12.9% (−34.6% to 8.8%)		3 (10.3)	11 (36.7)	−26.3% (−47.2% to −3.7%)	
Unchanged	24 (82.8)	22 (73.3)	9.4% (−12.7% to 31.5%)		24 (82.8)	18 (60)	22.8% (−1.8% to 44.8%)	
Improved	1 (3.4)	0 (0.0)	3.4% (−8.6% to 18.5%)		2 (6.9)	1 (3.3)	3.6% (−11.9% to 19.7%)	
Sick leave				.15				.32
Deteriorated	0 (0.0)	2 (6.7)	−6.7% (−22.1% to 6.0%)		1 (3.4)	4 (13.3)	−9.9% (−28.3% to 6.2%)	
Unchanged	16 (55.2)	19 (63.3)	−8.2% (−33.0% to 17.6%)		13 (44.8)	13 (43.3)	1.5% (−24.6% to 27.0%)	
Improved	13 (44.8)	9 (30)	14.8% (−10.6% to 38.9%)		15 (51.7)	13 (43.3)	8.4% (−18.5% to 33.7%)	
GSE^b^ score^c^				.29				.12
Deteriorated	4 (13.8)	7 (23.3)	−9.5% (−31.5% to 11.9%)		2 (6.9)	10 (33.3)	−26.4% (−47.2% to −5.5%)	
Unchanged	22 (75.9)	22 (73.3)	2.5% (−20.9% to 25.2%)		23 (79.3)	16 (53.3)	26.0% (1.3% to 48.6%)	
Improved	3 (10.3)	1 (3.3)	7.0% (−8.6% to 25.1%)		4 (13.8)	4 (13.3)	0.5% (−18.8% to 20.1%)	

a Missing data were imputed using last observation carried forward.

b GSE: general self-efficacy.

c ≥5 points was used as the threshold for minimal significant change.

There was a significant intergroup difference in the mean GSE score at the 6-month follow-up, with the intervention group having higher scores (*P*=.03; Table 3). Regarding the mean sick leave, there was a significant difference between the groups at the 3-month follow-up (*P*=.02; Table 3). Neither of these differences was present at baseline. Additionally, in the intervention group, sick leave significantly decreased from baseline to both the 3- and 6-month follow-ups (*P*<.001), a significance also found in the nonimputed analysis (Table S4 in Multimedia Appendix 3).

**Table 3.** Mean values for self-reported sick leave and general self-efficacy^a^.

Outcome	Intervention (n=29), mean (SD)	Control (n=30), mean (SD)	Change from baseline to 3- and 6-month follow-up					
			Mean difference within groups (95% CI)				Mean difference between groups (95% CI)	
			Intervention group (n=29)	*P* value	Control group (n=30)	*P* value	Intervention and control groups (n=59)	*P* value
GSE^b^								
Baseline	29.17 (6.84)	28.83 (5.05)	—^c^	—	—	—	—	—
3-month follow-up	29.62 (5.75)	27.37 (5.38)	0.52 (−1.08 to 2.11)	.52	−1.53 (−3.10 to 0.03)	.06	2.05 (−0.19 to 4.29)	.07
6-month follow-up	30.21 (5.95)	27.13 (6.21)	1.10 (−0.72 to 2.93)	.23	−1.77 (−3.56 to 0.03)	.05	2.87 (0.31 to 5.43)	.03
Sick leave								
Baseline	66.38 (33.59)	65.83 (37.42)	—	—	—	—	—	—
3-month follow-up	32.76 (37.86)	54.17 (45.05)	−33.48 (−47.07 to −19.88)	<.001	−11.81 (−24.27 to 0.66)	.06	−21.67 (−40.10 to −3.24)	.02
6-month follow-up	28.45 (38.80)	48.33 (43.52)	−37.79 (−52.21 to −23.36)	<.001	−17.64 (−32.62 to −2.66)	.02	−20.15 (−40.93 to 0.64)	.06

a Data were imputed using the last observation carried forward.

b GSE: general self-efficacy.

c Not available.

## Discussion

### Principal Findings

While previous studies have evaluated remotely delivered and/or person-centered interventions for persons with chronic pain, RCTs investigating the efficacy of remote person-centered interventions combining telephone support and eHealth platforms remain limited. Additionally, the present trial focused specifically on working-age individuals currently on sick leave due to nonmalignant chronic pain, with primary outcomes including self-efficacy and level of sick leave. Our results showed a statistically significant difference in the composite score for GSE and sick leave, favoring the intervention group receiving PCC, which may indicate a protective role of PCC.

In this study, the intervention group reported a mean increase in self-efficacy, while the control group reported a decline, rendering in a significant difference between the groups at the 6-month follow-up. Given the previously described reciprocal relationship between chronic pain and lower self-efficacy [28,45], we suggest that the decline in the GSE score shown by the control group may be considered part of the natural course for people living with chronic pain. Chronic pain can be a progressive condition, with exacerbations, worsening of symptoms over time, or resistance to initially effective treatments [46]. Thus, our findings suggest that PCC may stabilize outcomes or prevent decline over time, which can be of importance for the target population.

The core of PCC and, therefore, of the intervention is the partnership between the HCP and the patient [17,19], where the patient is treated as a person and supported in realizing their capabilities [47]. In addition, creating a health plan in partnership with the patient and the HCP, with emphasis on identifying the patient’s resources and capabilities, can support the patient in achieving their goals and thereby reinforce their self-efficacy [48]. Identifying and reinforcing a person’s resources align well with increasing self-efficacy, which could explain the intervention’s positive results. In previous PCC interventions, participants found the telephone support, followed by the jointly created health plans, to be most meaningful [49], indicating that these parts of the complex intervention may be important for the results. In PCC, a person’s resources are central, and because self-efficacy reflects the person’s belief in their ability to use these resources [23], GSE is often considered an appropriate outcome of PCC interventions. Furthermore, for people with chronic pain, reduced pain is not always a realistic or feasible goal. As Cohen et al [14] argued in their 2021 *Lancet* series on chronic pain, the goals of pain management should encompass other outcomes than reduction of pain intensity. From this perspective, self-efficacy may therefore be a meaningful outcome, and our RCT confirms previous recommendations that PCC has a pivotal role in supporting people with chronic pain [50] and should be part of their care [51].

Our results showed within-group decreases in mean sick leave from baseline to the 3-month follow-up for the intervention group and to the 6-month follow-up for both the intervention and control groups. This resulted in a significant between-group difference in sick leave level at the 3-month follow-up, a difference that did not persist at the 6-month follow-up. This result is still noteworthy. While both groups had comparable sick leave levels at baseline, the intervention group had a larger decrease after 6 months than the control group, suggesting that PCC has a clinically relevant impact on sick leave level. Through the intervention in this study, a noninvasive, nonpharmacological treatment, sick leave was more than halved in the intervention group in just 6 months. The results contrast with previous research that found that sick leave and return to work in persons with chronic pain can be hard to affect through health care interventions [52]. It is important for people with chronic pain to return to work [53], and being seen as a capable person is essential when receiving support [54]. In combination, this suggests that the intervention’s focus on self-efficacy and sick leave aligns with what the target population perceived as meaningful support and desirable goals.

Unlike previous remote PCC interventions in other study populations [21,22], the difference in the composite score in our study was not significant at 3 months but emerged at 6 months. This delay may reflect either implementation differences, affecting the time required for treatment initiation or adjustment to yield benefits, or varying patient needs. Patients with chronic pain may require more time to establish trust in a partnership with HCPs, as they often have negative experiences during encounters in health care [1]. Future research may benefit from further exploration of differences between patient groups, implementation processes and mechanisms, and long-term outcomes of the EAPER-P intervention.

Remote PCC may be especially beneficial, as it can improve patient participation as well as enhance the acquisition of self-efficacy and self-management strategies for the participants’ condition [55]. Remote interventions are often appreciated by persons with chronic pain, as they can be adjusted to the patients’ preferences regarding factors such as space, place, and pace of the intervention [55]. Additionally, they are often low-resource and easily scalable, which could enhance the integration of nonpharmacological pain treatment in health care [56] and appears to have no reported adverse effects [57]. The PCC intervention in this study, with its accessible format, delivered regardless of location and tailored to the persons’ preferences, resources, and goals, has the potential to be delivered by HCPs in primary care, and further studies are warranted to explore its implementation in usual care.

### Methodological Considerations

This study has some limitations worth mentioning. First, the study did not reach the number of participants calculated in the a priori power calculation. The challenges of recruiting persons living with chronic pain are well known and have been reported elsewhere [58,59]. Despite this, the study showed statistically significant differences between the groups at the end point in the composite score, combining GSE and sick leave. The sensitivity analyses confirmed that the statistically significant differences were maintained in the analyses based on the nonimputed data. However, future larger-scale studies are warranted to confirm the findings observed in this trial.

Second, the intervention was evaluated in an efficacy trial and was therefore administered in a research context under ideal conditions, for example, with HCPs dedicated solely to working on the study. This factor may impact the transferability of the results to a clinical setting. However, measuring efficacy instead of effectiveness often means that internal validity is higher and that efficacy can be estimated with less bias [60]. In a clinical setting, interventions can also lead to spillover effects, where outcomes extend beyond those intended, especially when HCPs are involved in multiple activities, treating multiple groups, moving between units, or sharing common social areas [61].

Third, using self-reported data for the outcomes can be problematic. There is a risk of inaccurate reporting regarding sick leave [62], and questionnaires may be misinterpreted. However, sick leave records do not always correspond to the actual level of sick leave, as people may choose to go back to work earlier without the records being updated instantly. Also, some may not go back to work even if the sick leave level is reported as reduced.

Fourth, a potential limitation of this study is the lack of blinding. Unfortunately, blinding was not feasible given the nature of the intervention, which required active participant involvement and interaction with HCPs. Hence, participants in the intervention group did receive additional structured attention that the control group did not, introducing the risk of performance bias that might have contributed to improvements not driven by the intervention’s components. Future studies could aim to minimize this bias to better isolate the efficacy of the intervention, for example, by adding an attention-matched control group.

The study also has some methodological strengths. One strength of the intervention is that a third of the study population was born outside Sweden, which closely reflects the population living in the region [63]. Having a diverse participant population is important, as country of birth has been shown to affect the severity of clinical presentations of pain as well as outcomes of pain rehabilitation [64]. This could therefore enhance the generalizability of the results.

Another strength of the intervention is that it was carried out entirely remotely through telephone calls and an eHealth platform. Previous research indicates that participants perceived a similarly designed remote format as meaningful because it reduced barriers to accessing support and fostered a sense that support was readily available when needed [49], and that direct-dial telephone access explained meaningful use among participants with lower technical competence [65]. Additionally, people with chronic pain often report experiences of immobility, such as difficulty walking or driving a car [1]. Another barrier to participating in on-site treatment can be fatigue. This can be a greater obstacle in daily life than even the pain itself [66]. The remoteness of the intervention made the treatment accessible, which could have contributed to the study’s high retention, with no dropouts.

### Conclusions

This study suggests that a PCC intervention delivered via telephone and an eHealth platform may influence the level of sick leave and self-efficacy among persons with chronic pain. Our study contributes to the growing evidence supporting the importance of PCC in people with chronic pain by increasing self-efficacy, which may promote self-management strategies and affect sick leave from work. Further studies are warranted to confirm the findings observed in this trial.

## Supplementary material

10.2196/91887 Multimedia Appendix 1 EAPER-P (Early Accessible Person-Centered Rehabilitation for Patients With Chronic Pain) statistical analysis plan.

10.2196/91887 Multimedia Appendix 2 Prespecified medical history and concomitant medications.

10.2196/91887 Multimedia Appendix 3 Composite, self-reported sick leave and general self-efficacy (GSE) score at 3 and 6 months postinclusion and mean self-reported sick leave and GSE scores. No imputation of data was performed.

10.2196/91887 Checklist 1 CONSORT-eHEALTH checklist (V 1.6.1).

10.2196/91887 Checklist 2 GRIPP2-Short Form checklist.

## Abbreviations

CONSORT-EHEALTH: Consolidated Standards of Reporting Trials of Electronic and Mobile Health Applications and Online Telehealth
EAPER-P: Early Accessible Person-Centered Rehabilitation for Patients With Chronic Pain
GRIPP-SF: Guidance for Reporting Involvement of Patients and the Public-Short Form
GSE: general self-efficacy
HCP: health care professional
*ICD-10*: *International Statistical Classification of Diseases and Related Health Problems, 10th Revision*
PCC: person-centered care
RCT: randomized controlled trial
