# Supplementary material for: Efficacy of a Remote Person-Centered Intervention Using an eHealth Platform and Telephone Support for Persons With Chronic Pain: Randomized Controlled Trial
Source: JMIR Form Res. 2026 Aug 3;10:e91887. doi: 10.2196/91887 (PMC13432249; doi:10.2196/91887)
Supplement: Multimedia Appendix 1 [file formative-v10-e91887-s001.pdf]

APNC
Statistics in Life Science

Statistical Analysis Plan

EAPER - Manuscript

May 21, 2025

Authored by:
Christopher Backström, Study Statistician

Approved by:
Aldina Pivodic, Senior Statistician
Sara Wallström, Principal Investigator

Revisions:

Nr	Description	Date
1	First draft.	20250521

Table of Contents

1 Background 4
2 Study Objectives and Hypothesis 4
2.1 Primary Objective and Hypothesis 4
3 Study Design 4
3.1 Treatment Groups/Exposure 4
4 Study Populations 4
4.1 Full Analysis Set/Intention-to-Treat Population 4
5 Study Variables 4
5.1 Baseline/Patient Characteristics Variables 4
5.1.1 Demographics and Baseline Characteristics 4
5.2 Concomitant Medications 5
5.3 Efficacy/Outcome Variables 5
5.3.1 Primary Variable 5
5.3.2 Secondary Variables 5
6 Statistical Methods 6
6.1 Sample Size 6
6.2 General Methodology 6
6.3 Adjustment for Type I Error 7
6.4 Handling of Missing Data 7
7 Statistical Analyses 7
7.1 Demographics and Baseline Characteristics 7
7.2 Concomitant Medications 7
7.3 Efficacy Analyses 7
7.3.1 Primary Efficacy Analysis 7
7.3.2 Secondary Efficacy Analyses 7
8 Planned Tables and Figures 8

Abbreviations

**ACE**	Angiotensin converting enzyme
**AE**	Adverse events
**ANCOVA**	Analysis of covariance
**ASA**	Acetylsalicylic acid
**ATC**	Anatomical therapeutic classification
**CABG**	Coronary artery bypass grafting
**CAT**	COPD assessment test
**CHF**	Chronic heart failure
**CI**	Confidence interval
**COPD**	Chronic obstructive pulmonary disease
**CRF**	Case report form
**CRT**	Cardiac resynchronisation therapy
**GSE**	General self-efficacy scale
**HADS**	Hospital anxiety and depression scale
**HR**	Hazard ratio
**ICD**	International classification of diseases
**KM**	Kaplan Meier
**MID**	Minimal important difference
**mMRC**	Modified Medical research council
**PCC**	Person-centred care
**PT**	Preferred term
**SAP**	Statistical analysis plan
**SD**	Standard deviation
**SOB-HF**	Shortness of breath in heart failure
**SOC**	System organ class

1 Background

The aim of this project is to evaluate if person-centered care (PCC) in the form of a combined eHealth and structured telephone support available at home will improve general self-efficacy and reduce self-reported sick leave for patients with chronic pain.

This statistical analysis plan (SAP) is describing the study's quantitative objectives and planned analyses for the study's main manuscript. The qualitative part, other quantitative sub-studies, and health economic evaluations will be planned in a separate document.

2 Study Objectives and Hypothesis

2.1 Primary Objective and Hypothesis

Does a person-centred intervention via a combined telephone and e-health support for people with chronic pain, in comparison with a control group, lead to:

- increased self-efficacy?
- reduced rate of self-reported sick leave?

The hypothesis is that PCC increases self-efficacy and reduces rate of self-reported sick leave.

3 Study Design

3.1 Treatment Groups/Exposure

The treatment groups will be called as following:

- Intervention
- Control

4 Study Populations

4.1 Full Analysis Set/Intention-to-Treat Population

All randomized patients will be included in the Intention-to-Treat (ITT) population.
All ITT summaries will be performed on as-randomized groups.

5 Study Variables

5.1 Baseline/Patient Characteristics Variables

Baseline is defined at the date of randomisation and counted as day 1 in study.

5.1.1 Demographics and Baseline Characteristics

Following demographics and baseline characteristics will be described:

- Age (years)
- Sex (Male, Female)
- Diagnosis (M25, M54, M79, R52)
- Living situation (Married/living together, Living apart, Single)
- Education (Compulsory school or lower, Upper secondary or high school, Post-secondary vocational school, College/University)
- Country of birth (Sweden, Other)

- Pre-specified medical history
    - Depression (F32)
    - Anxiety (F41)
    - Exhaustion disorder /stress (F43)
    - Insomnia (G43)
    - Migraine (G43)
    - Hyperactivity disorder (F90)

5.2 Concomitant Medications

Use of following pre-specified medications will be described:

- Opioids (N02A)
- Antidepressants (N06A)
- NSAID/ Coxibes (M01AB, M01AE, M01AH, M02AA)
- Gabapentinoids (N03AX)
- Capsaicine (N01BX04)
- Karbamazepin (N03AF01)
- Sedatives (N05A/B, R06AD1/2,R06AD52)
- Muscle relaxants (M03BB03, M03BC, M03BX01)
- Sleep medication (N05C)
- Other analgetics, antipyretics, anesthetics (N02BA01, N02BA51, N02BE01, N01BB02)
- Other (C02AC01)

5.3 Efficacy/Outcome Variables

5.3.1 Primary Variable

In line with previous RCTs evaluating PCC the primary outcome will be a composite comprised of general self-efficacy and self-reported sick leave after 6 months. Each participant will be classified as improved, deteriorated, or unchanged at six months as follows:

- Improved is a participant who has a reduced degree of self-reported sick leave and an increased self-efficacy by ≥5 units, which is seen as a clinically relevant improvement.
- Deteriorated is a participant who has an increased degree of self-reported sick leave or a decrease in self-efficacy by ≥ 5 units.
- The participants who are neither improved nor impaired are counted as unchanged.

In case of missing data the last available data will be carried forward if available, otherwise unchanged value will be imputed that is corresponding to baseline data carried forward.

5.3.2 Secondary Variables

Following secondary efficacy variables will be analysed:

- Composite comprised of general self-efficacy and self-reported sick leave (as 'Deteriorated', 'Unchanged' and 'Improved') after 3 months.
- Degree of sick leave at 3, 6 months. In case of missing data, the last available data will be used from baseline or post-baseline data collection:
  - Change in sick leave (as continuous variable)
  - Change in sick leave (as the categorical variable 'Deteriorated', 'Unchanged' and 'Improved')
- Change in GSE at 3, 6 months from baseline. In case of missing data, the last available data will be used from baseline or post-baseline data collection. GSE will be analysed through the following two variables:
  - Change in GSE (as continuous variable)
  - Change in GSE by ≥5 units (as the categorical variable 'Deteriorated', 'Unchanged' and 'Improved')

6 Statistical Methods

6.1 Sample Size

To achieve 80% power based on a p-value of 0.05 (two-tailed) for an increase in the proportion of improved patients from 20 to 40% in the primary outcome a sample size of 91 patients in each group was required. However, the study team planned to include 110 patients in each group to account for withdrawals/dropouts. Thus, a minimum of 220 patients were planned to be randomised across the two groups.

6.2 General Methodology

The first analysis report will be performed using data up to 6 months that is the time point used in the primary analysis. Six months data will be analysed as main analyses and 3 months data as complementary.

Descriptively, continuous variables will be presented by mean, standard deviation (SD), median, minimum and maximum, and categorical variables by number and percentages.

For all efficacy analyses the relevant estimate will be presented for comparison between the groups, difference in means or/and proportions, accompanied by 95% confidence intervals (CI).

For test between the two groups with respect to dichotomous variables Fisher's exact test will be used, for ordered categorical variables Mantel-Haenszel chi-square test will be used, non-ordered categorical variables chi-square test will be used and for continuous variables T-test or Mann-Whitney U-test will be used.

The change in continuous variables over time will be evaluated by using mixed models for repeated measures using unstructured covariance matrix and adjusting for baseline value. Mean difference with 95% CI will be described along with p-values within and between treatment groups.

The primary analysis will be considered confirmed if the p-value<0.05 could be achieved. All other analyses will be considered as exploratory and the p-values will be interpreted as descriptive.

Figures of continuous variables over the follow-up period and change from baseline over the follow-up period will be given as box-plots and figures of dichotomous and ordered categorical variables over the follow-up period and change from baseline over the follow-up period will be given as vertical bar charts.

Missing data will be handled in the definition as primary and secondary variables. See section 5.3.1 and 5.3.2. No other missing data will be imputed.

Additional analyses will be performed using only non-imputed data.

All tests will be two-tailed and conducted at 0.05 significance level, following adjustments as described.

All analyses will be performed using SAS software version 9.4 (SAS Institute Inc. Cary, NC, USA).

6.3 Adjustment for Type I Error

No adjustment for type I error will be performed. The primary analysis will be considered confirmed if the p-value<0.05 is achieved. All other tests will be exploratory.

6.4 Handling of Missing Data

Missing data will be handled as provided in the definition of the primary and secondary variables. See section 5.3.1 and 5.3.2. No other missing data will be imputed.

7 Statistical Analyses

7.1 Demographics and Baseline Characteristics

Demographics and baseline characteristics will be summarized by treatment group for the ITT population and analysed according to the methods described in section "General Methodology" above.

7.2 Concomitant Medications

Concomitant medication will be summarised by treatment group for ITT population.

7.3 Efficacy Analyses

7.3.1 Primary Efficacy Analysis

The primary efficacy variable, the composite of change in the General Self-Efficacy (GSE) scale, degree of self-reported sick leave, evaluated at 6 months follow-up after baseline will be analysed as ordered categorical, with the categories: 'Deteriorated', 'Unchanged' and 'Improved'. The primary variable will be analysed using Mantel-Haenszel chi-square trend test. The difference of proportions between the treatments for each category will be described along with the 95% CI for proportions.

A sensitivity analysis will be performed on the primary outcome without imputation.

Graphically, the categories of the primary variable will be presented using histograms.

7.3.2 Secondary Efficacy Analyses

The secondary efficacy analyses will be performed according to the general methodology above.

8 Planned Tables and Figures

Table Number	Table Title
**Table 1.**	Demographics and baseline characteristics for intervention and control group. Data are presented as mean ± standard deviation, median (range) and number of observations, or number (percentage) (ITT population).
**Table 2.**	Composite, self-reported sick leave and GSE at 3 and 6 months. Data imputed with Last Observation Carried Forward (LOCF) (ITT population).
**Table 3.**	Mean values self-reported sick leave and GSE. Data imputed with Last Observation Carried Forward (LOCF) (ITT population).
**Supplementary table 1.**	Pre-specified medical history and concomitant medication for intervention and control group.
**Supplementary table 2.**	Composite, self-reported sick leave and GSE at 3 and 6 months. No imputation of data (ITT population).
**Supplementary table 3.**	Mean values of self-reported sick leave and GSE. No imputation of data (ITT population).

Figure Number	Figure Title
**Figure 1**	Change in the composite endpoint from baseline over the follow-up period by randomized groups, vertical bar-charts (ITT population).
**Figure 2.1**	Change in self-reported sick leave from baseline over the follow-up period by randomized groups, box-plots (ITT population).
**Figure 2.2**	Change in GSE from baseline over the follow-up period by randomized groups, box-plots (ITT population).
**Figure 3.1**	Change in sick leave from baseline over the follow-up period by randomized groups, vertical bar-charts (ITT population).
**Figure 3.2**	Change in GSE ≥ 5 units from baseline over the follow-up period by randomized groups, vertical bar-charts (ITT population).
