# Supplementary material for: Efficacy of a Remote Person-Centered Intervention Using an eHealth Platform and Telephone Support for Persons With Chronic Pain: Randomized Controlled Trial
Source: JMIR Form Res. 2026 Aug 3;10:e91887. doi: 10.2196/91887 (PMC13432249; doi:10.2196/91887)
Supplement: Multimedia Appendix 2 [file formative-v10-e91887-s002.docx]

Table S1. Pre-specified medical history.

| **Variable (ICD-10)** | **Intervention group**  **n=29** | **Control group**  **n=30** |
| --- | --- | --- |
| **Depression (F32)** |  |  |
| Yes | 5 (17.2%) | 8 (26.7%) |
| No | 24 (82.8%) | 22 (73.3%) |
| **Anxiety (F41)** |  |  |
| Yes | 11 (37.9%) | 7 (23.3%) |
| No | 18 (62.1%) | 23 (76.7%) |
| **Exhaustion disorder/stress (F43)** |  |  |
| Yes | 12 (41.4%) | 10 (33.3%) |
| No | 17 (58.6%) | 20 (66.7%) |
| **Insomnia (G43)** |  |  |
| Yes | 8 (27.6%) | 7 (23.3%) |
| No | 21 (72.4%) | 23 (76.7%) |
| **Migraine (G43)** |  |  |
| Yes | 3 (10.3%) | 3 (10.0%) |
| No | 26 (89.7%) | 27 (90.0%) |
| **Endometriosis (N80)** |  |  |
| Yes | 0 (0.0%) | 2 (6.7%) |
| No | 29 (100.0%) | 28 (93.3%) |
| **Hyperactivity disorder (F90)** |  |  |
| Yes | 0 (0.0%) | 1 (3.3%) |
| No | 29 (100.0%) | 29 (96.7%) |

Table S2. Concomitant medications.

| **Concomitant medications (ATC)** | **Intervention group n=29** | **Control group n=30** |  |
| --- | --- | --- | --- |
| **Opioids (N02A)** |  |  |  |
| Yes | 10 (34.5%) | 7 (23.3%) |  |
| No | 19 (65.5%) | 23 (76.7%) |  |
| **Antidepressants (N06A)** |  |  |  |
| Yes | 12 (41.4%) | 8 (26.7%) |  |
| No | 17 (58.6%) | 22 (73.3%) |  |
| **NSAIDs/Coxibes (M01AB, M01AE, M01AH, M02AA)** |  |  |  |
| Yes | 18 (62.1%) | 17 (56.7%) |  |
| No | 11 (37.9%) | 13 (43.3%) |  |
| **Gabapentinoids (N03AX)** |  |  |  |
| Yes | 3 (10.3%) | 7 (23.3%) |  |
| No | 26 (89.7%) | 23 (76.7%) |  |
| **Capsaicin (N01BX04)** |  |  |  |
| No | 29 (100.0%) | 30 (100.0%) |  |
| **Carbamazepin (N03AF01)** |  |  |  |
| Yes | 1 (3.4%) | 0 (0.0%) |  |
| No | 28 (96.6%) | 30 (100.0%) |  |
| **Sedatives (N05A/B, R06AD1/2,R06AD52)** |  |  |  |
| Yes | 5 (17.2%) | 2 (6.7%) |  |
| No | 24 (82.8%) | 28 (93.3%) |  |
| **Muscle relaxants (M03BB03, M03BC, M03BX01)** |  |  |  |
| Yes | 4 (13.8%) | 11 (36.7%) |  |
| No | 25 (86.2%) | 19 (63.3%) |  |
| **Sleep medication (N05C)** |  |  |  |
| Yes | 4 (13.8%) | 8 (26.7%) |  |
| No | 25 (86.2%) | 22 (73.3%) |  |
| **Other analgesics, antipyretics, anaesthetics (N02BA01, N02BA51, N02BE01, N01BB02)** |  |  |  |
| Yes | 14 (48.3%) | 13 (43.3%) |  |
| No | 15 (51.7%) | 17 (56.7%) |  |
| **Other (C02AC01)** |  |  |  |
| No | 29 (100.0%) | 30 (100.0%) |  |
| ATC: anatomical therapeutic chemical classification system | | | |
| NSAID: nonsteroidal anti-inflammatory drugs | | | |
