# Supplementary material for: Efficacy of a Remote Person-Centered Intervention Using an eHealth Platform and Telephone Support for Persons With Chronic Pain: Randomized Controlled Trial
Source: JMIR Form Res. 2026 Aug 3;10:e91887. doi: 10.2196/91887 (PMC13432249; doi:10.2196/91887)
Supplement: Multimedia Appendix 3 [file formative-v10-e91887-s003.docx]

Table S3. Composite, self-reported sick leave and General Self-Efficacy (GSE) score at 3- and 6-months post-inclusion. No imputation of data was performed.

|  | **3-month follow-up** | | | | **6-month follow-up** | | | |
| --- | --- | --- | --- | --- | --- | --- | --- | --- |
|  | **Intervention group**  **n=29** | **Control group**  **n=30** | **Difference in proportion**  **(95% CI)** | ***P*** | **Intervention group**  **n=29** | **Control group**  **n=30** | **Difference in proportion**  **(95% CI)** | ***P*** |
| **Composite** |  |  |  | .122 |  |  |  | .035 |
| Deteriorated | 4 (17.4%) | 8 (38.1%) | -20.7% (-47.6%; 6.6%) |  | 3 (13.0%) | 11 (44.0%) | -31.0% (-54.5%; -4.4%) |  |
| Unchanged | 18 (78.3%) | 13 (61.9%) | 16.4% (-11.6%; 43.7%) |  | 18 (78.3%) | 13 (52.0%) | 26.3% (-1.4%; 51.5%) |  |
| Improved | 1 (4.3%) | 0 (0.0%) | 4.3% (-12.7%; 22.6%) |  | 2 (8.7%) | 1 (4.0%) | 4.7% (-13.9%; 24.6%) |  |
| Missing | *6* | *9* |  |  | *6* | *5* |  |  |
| **Sick leave** |  |  |  | .215 |  |  |  | .403 |
| Deteriorated | 0 (0.0%) | 2 (9.1%) | -9.1% (-29.2%; 6.1%) |  | 1 (4.2%) | 4 (15.4%) | -11.2% (-31.0%; 7.7%) |  |
| Unchanged | 11 (45.8%) | 11 (50.0%) | -4.2% (-33.0%; 25.4%) |  | 9 (37.5%) | 9 (34.6%) | 2.9% (-24.3%; 29.6%) |  |
| Improved | 13 (54.2%) | 9 (40.9%) | 13.3% (-16.4%; 41.5%) |  | 14 (58.3%) | 13 (50.0%) | 8.3% (-19.9%; 36.0%) |  |
| Missing | *5* | *8* |  |  | *5* | *4* |  |  |
| **GSE*** |  |  |  | .194 |  |  |  | .166 |
| Deteriorated | 4 (17.4%) | 7 (31.8%) | -14.4% (-40.8%; 11.7%) |  | 2 (8.7%) | 10 (38.5%) | -29.8% (-52.2%; -4.8%) |  |
| Unchanged | 16 (69.6%) | 14 (63.6%) | 5.9% (-22.3%; 33.6%) |  | 18 (78.3%) | 12 (46.2%) | 32.1% (3.9%; 57.2%) |  |
| Improved | 3 (13.0%) | 1 (4.5%) | 8.5% (-11.4%; 29.2%) |  | 3 (13.0%) | 4 (15.4%) | -2.3% (-24.1%; 20.2%) |  |
| Missing | *6* | *8* |  |  | *6* | *4* |  |  |
| CI = confidence interval | | | | | | | | |
| GSE = general self-efficacy | | | | | | | | |
| SD = standard deviation | | | | | | | | |
| *≥5 points was used as the threshold for minimal significant change | | | | | | | | |

Table S4. Mean self-reported sick leave and General Self-Efficacy (GSE) scores. No imputation of data was performed.

|  | | | | **Change from baseline to 3- and 6-months follow-up** | | | | | |
| --- | --- | --- | --- | --- | --- | --- | --- | --- | --- |
|  | | **Mean ±SD** | | **Mean difference within groups (95% CI)** | | | | **Mean difference between groups (95% CI)** | |
| **Outcome** | **Visit** | **Intervention group**  **n=29** | **Control group**  **n=30** | **Intervention group**  **n=29** | ***P*** | **Control group**  **n=30** | ***P*** | **Intervention & control group =59** | ***P*** |
| GSE | Baseline | 29.17±6.84 | 28.83±5.05 |  |  |  |  |  |  |
|  | 3-month follow-up | 29.39±5.96 | 27.50±5.26 | 0.43 (-1.55; 2.40) | .665 | -1.67 (-3.68; 0.33) | .100 | 2.10 (-0.71; 4.92) | .140 |
|  | 6-month follow-up | 29.52±5.62 | 27.50±6.11 | 0.64 (-1.41; 2.70) | .533 | -1.80 (-3.73; 0.13) | .068 | 2.44 (-0.38; 5.26) | .088 |
| Sick leave | Baseline | 66.38±33.59 | 65.83±37.42 |  |  |  |  |  |  |
|  | 3-month follow-up | 27.08±35.29 | 40.22±42.47 | -38.96 (-52.95; -24.97) | <0.001 | -18.59 (-34.02; -3.16) | .019 | -20.37 (-41.11; 0.37) | .054 |
|  | 6-month follow-up | 21.88±35.59 | 42.59±42.07 | -42.32 (-57.12; -27.53) | <0.001 | -23.26 (-38.24; -8.28) | .003 | -19.06 (-40.23; 2.11) | .077 |
| CI = confidence interval | | | | | | | | | |
| GSE = general self-efficacy | | | | | | | | | |
| SD = standard deviation | | | | | | | | | |
