# Supplementary material for: Efficacy of a Remote Person-Centered Intervention Using an eHealth Platform and Telephone Support for Persons With Chronic Pain: Randomized Controlled Trial
Source: JMIR Form Res. 2026 Aug 3;10:e91887. doi: 10.2196/91887 (PMC13432249; doi:10.2196/91887)
Supplement: Checklist 1 [file formative-v10-e91887-s004.pdf]

CONSORT-EHEALTH (V 1.6.1) - Submission/Publication Form

The CONSORT-EHEALTH checklist is intended for authors of randomized trials evaluating web-based and Internet-based applications/interventions, including mobile interventions, electronic games (incl multiplayer games), social media, certain telehealth applications, and other interactive and/or networked electronic applications. Some of the items (e.g. all subitems under item 5 - description of the intervention) may also be applicable for other study designs.

The goal of the CONSORT EHEALTH checklist and guideline is to be
a) a guide for reporting for authors of RCTs,
b) to form a basis for appraisal of an ehealth trial (in terms of validity)

CONSORT-EHEALTH items/subitems are MANDATORY reporting items for studies published in the Journal of Medical Internet Research and other journals / scientific societies endorsing the checklist.

Items numbered 1., 2., 3., 4a., 4b etc are original CONSORT or CONSORT-NPT (non-pharmacologic treatment) items.
Items with Roman numerals (i., ii, iii, iv etc.) are CONSORT-EHEALTH extensions/clarifications.

As the CONSORT-EHEALTH checklist is still considered in a formative stage, we would ask that you also RATE ON A SCALE OF 1-5 how important/useful you feel each item is FOR THE PURPOSE OF THE CHECKLIST and reporting guideline (optional).

Mandatory reporting items are marked with a red *.
In the textboxes, either copy & paste the relevant sections from your manuscript into this form - please include any quotes from your manuscript in QUOTATION MARKS,
or answer directly by providing additional information not in the manuscript, or elaborating on why the item was not relevant for this study.

YOUR ANSWERS WILL BE PUBLISHED AS A SUPPLEMENTARY FILE TO YOUR PUBLICATION IN JMIR AND ARE CONSIDERED PART OF YOUR PUBLICATION (IF ACCEPTED).
Please fill in these questions diligently. Information will not be copyedited, so please use proper spelling and grammar, use correct capitalization, and avoid abbreviations.

DO NOT FORGET TO SAVE AS PDF _AND_ CLICK THE SUBMIT BUTTON SO YOUR ANSWERS ARE IN OUR DATABASE !!!

Citation Suggestion (if you append the pdf as Appendix we suggest to cite this paper in the caption):
Eysenbach G, CONSORT-EHEALTH Group
CONSORT-EHEALTH: Improving and Standardizing Evaluation Reports of Web-based and Mobile Health Interventions
J Med Internet Res 2011;13(4):e126

URL: http://www.jmir.org/2011/4/e126/
doi: 10.2196/jmir.1923
PMID: 22209829

ase.lundin@gu.se Byt konto

Inte delad

Utkastet har sparats

* Anger obligatorisk fråga

Your name *

First Last

Åse Lundin

Primary Affiliation (short), City, Country *

University of Toronto, Toronto, Canada

University of Gothenburg, Gothenburg, Sweden

Your e-mail address *

abc@gmail.com

ase.lundin@gu.se

Title of your manuscript *

Provide the (draft) title of your manuscript.

Efficacy of a Remote Person-centred Intervention Using an eHealth Platform and Telephone Support for Persons with Chronic Pain: A Randomized Controlled Trial

Name of your App/Software/Intervention *

If there is a short and a long/alternate name, write the short name first and add the long name in brackets.

MyHealth

Evaluated Version (if any)

e.g. "V1", "Release 2017-03-01", "Version 2.0.27913"

V 1.6.1

Language(s) *

What language is the intervention/app in? If multiple languages are available, separate by comma (e.g. "English, French")

Swedish

URL of your Intervention Website or App

e.g. a direct link to the mobile app on app in appstore (itunes, Google Play), or URL of the website. If the intervention is a DVD or hardware, you can also link to an Amazon page.

https://minhalsa.smarta.it.gu.se/forskningsperson/home

URL of an image/screenshot (optional)

Ditt svar

Accessibility *

Can an enduser access the intervention presently?

- ○ access is free and open
- ○ access only for special usergroups, not open
- ○ access is open to everyone, but requires payment/subscription/in-app purchases
- ○ app/intervention no longer accessible
- ◉ Övrigt: Access is only for study participants and health professionals in the F

Primary Medical Indication/Disease/Condition *

e.g. "Stress", "Diabetes", or define the target group in brackets after the condition, e.g. "Autism (Parents of children with)", "Alzheimers (Informal Caregivers of)"

"Chronic pain" i.e. pain >3 months

Primary Outcomes measured in trial *

comma-separated list of primary outcomes reported in the trial

General self-efficacy, self-reported sick leave

Secondary/other outcomes

Are there any other outcomes the intervention is expected to affect?

Ditt svar

Recommended "Dose" *

What do the instructions for users say on how often the app should be used?

- ○ Approximately Daily
- ○ Approximately Weekly
- ○ Approximately Monthly
- ○ Approximately Yearly
- ◉ "as needed"
- ○ Övrigt:

Approx. Percentage of Users (starters) still using the app as recommended after 3 months *

- ◉ unknown / not evaluated
- ○ 0-10%
- ○ 11-20%
- ○ 21-30%
- ○ 31-40%
- ○ 41-50%
- ○ 51-60%
- ○ 61-70%
- ○ 71%-80%
- ○ 81-90%
- ○ 91-100%
- ○ Övrigt:

Overall, was the app/intervention effective? *

- (x) yes: all primary outcomes were significantly better in intervention group vs control
- ( ) partly: SOME primary outcomes were significantly better in intervention group vs control
- ( ) no statistically significant difference between control and intervention
- ( ) potentially harmful: control was significantly better than intervention in one or more outcomes
- ( ) inconclusive: more research is needed
- ( ) Övrigt:

Article Preparation Status/Stage *

At which stage in your article preparation are you currently (at the time you fill in this form)

- ( ) not submitted yet - in early draft status
- (x) not submitted yet - in late draft status, just before submission
- ( ) submitted to a journal but not reviewed yet
- ( ) submitted to a journal and after receiving initial reviewer comments
- ( ) submitted to a journal and accepted, but not published yet
- ( ) published
- ( ) Övrigt:

Journal *

If you already know where you will submit this paper (or if it is already submitted), please provide the journal name (if it is not JMIR, provide the journal name under "other")

- ○ not submitted yet / unclear where I will submit this
- ◉ Journal of Medical Internet Research (JMIR)
- ○ JMIR mHealth and UHealth
- ○ JMIR Serious Games
- ○ JMIR Mental Health
- ○ JMIR Public Health
- ○ JMIR Formative Research
- ○ Other JMIR sister journal
- ○ Övrigt:

Is this a full powered effectiveness trial or a pilot/feasibility trial? *

- ◉ Pilot/feasibility
- ○ Fully powered

Manuscript tracking number *

If this is a JMIR submission, please provide the manuscript tracking number under "other" (The ms tracking number can be found in the submission acknowledgement email, or when you login as author in JMIR. If the paper is already published in JMIR, then the ms tracking number is the four-digit number at the end of the DOI, to be found at the bottom of each published article in JMIR)

- ◉ no ms number (yet) / not (yet) submitted to / published in JMIR
- ○ Övrigt:

TITLE AND ABSTRACT

1a) TITLE: Identification as a randomized trial in the title

1a) Does your paper address CONSORT item 1a? *

I.e does the title contain the phrase "Randomized Controlled Trial"? (if not, explain the reason under "other")

- (●) yes
- ( ) Övrigt:

1a-i) Identify the mode of delivery in the title

Identify the mode of delivery. Preferably use “web-based” and/or “mobile” and/or “electronic game” in the title. Avoid ambiguous terms like “online”, “virtual”, “interactive”. Use “Internet-based” only if Intervention includes non-web-based Internet components (e.g. email), use “computer-based” or “electronic” only if offline products are used. Use “virtual” only in the context of “virtual reality” (3-D worlds). Use “online” only in the context of “online support groups”. Complement or substitute product names with broader terms for the class of products (such as “mobile” or “smart phone” instead of “iphone”), especially if the application runs on different platforms.

	1	2	3	4	5	
subitem not at all important	( )	( )	( )	(●)	( )	essential

Rensa markering

Does your paper address subitem 1a-i? *

Copy and paste relevant sections from manuscript title (include quotes in quotation marks "like this" to indicate direct quotes from your manuscript), or elaborate on this item by providing additional information not in the ms, or briefly explain why the item is not applicable/relevant for your study

Yes "Efficacy of a Remote Person-centred Intervention Using an eHealth Platform and Telephone Support for Persons with Chronic Pain: A Randomized Controlled Trial"

1a-ii) Non-web-based components or important co-interventions in title

Mention non-web-based components or important co-interventions in title, if any (e.g., “with telephone support”).

	1	2	3	4	5	
subitem not at all important	○	○	○	◉	○	essential

Rensa markering

Does your paper address subitem 1a-ii?

Copy and paste relevant sections from manuscript title (include quotes in quotation marks "like this" to indicate direct quotes from your manuscript), or elaborate on this item by providing additional information not in the ms, or briefly explain why the item is not applicable/relevant for your study

Yes "Efficacy of a Remote Person-centred Intervention Using an eHealth Platform and Telephone Support for Persons with Chronic Pain: A Randomized Controlled Trial"

1a-iii) Primary condition or target group in the title

Mention primary condition or target group in the title, if any (e.g., “for children with Type I Diabetes”) Example: A Web-based and Mobile Intervention with Telephone Support for Children with Type I Diabetes: Randomized Controlled Trial

	1	2	3	4	5	
subitem not at all important	○	○	○	◉	○	essential

Rensa markering

Does your paper address subitem 1a-iii? *

Copy and paste relevant sections from manuscript title (include quotes in quotation marks "like this" to indicate direct quotes from your manuscript), or elaborate on this item by providing additional information not in the ms, or briefly explain why the item is not applicable/relevant for your study

Yes, "Efficacy of a Remote Person-centred Intervention Using an eHealth Platform and Telephone Support for Persons with Chronic Pain: A Randomized Controlled Trial"

1b) ABSTRACT: Structured summary of trial design, methods, results, and conclusions

NPT extension: Description of experimental treatment, comparator, care providers, centers, and blinding status.

1b-i) Key features/functionalities/components of the intervention and comparator in the METHODS section of the ABSTRACT

Mention key features/functionalities/components of the intervention and comparator in the abstract. If possible, also mention theories and principles used for designing the site. Keep in mind the needs of systematic reviewers and indexers by including important synonyms. (Note: Only report in the abstract what the main paper is reporting. If this information is missing from the main body of text, consider adding it)

	1	2	3	4	5	
subitem not at all important	○	○	○	◉	○	essential

Rensa markering

Does your paper address subitem 1b-i? *

Copy and paste relevant sections from the manuscript abstract (include quotes in quotation marks "like this" to indicate direct quotes from your manuscript), or elaborate on this item by providing additional information not in the ms, or briefly explain why the item is not applicable/relevant for your study

Yes, "Methods: A two-armed, non-blinded randomized controlled trial was conducted. Both groups received usual care; the intervention group additionally participated in a PCC intervention via telephone and an eHealth platform. The primary outcome was a composite score consisting of change in general self-efficacy and self-reported sick leave at 6-month follow-up."

1b-ii) Level of human involvement in the METHODS section of the ABSTRACT

Clarify the level of human involvement in the abstract, e.g., use phrases like “fully automated” vs. “therapist/nurse/care provider/physician-assisted” (mention number and expertise of providers involved, if any). (Note: Only report in the abstract what the main paper is reporting. If this information is missing from the main body of text, consider adding it)

	1	2	3	4	5	
subitem not at all important	○	○	◉	○	○	essential

Rensa markering

Does your paper address subitem 1b-ii?

Copy and paste relevant sections from the manuscript abstract (include quotes in quotation marks "like this" to indicate direct quotes from your manuscript), or elaborate on this item by providing additional information not in the ms, or briefly explain why the item is not applicable/relevant for your study

No, not in abstract due to word limit

1b-iii) Open vs. closed, web-based (self-assessment) vs. face-to-face assessments in the METHODS section of the ABSTRACT

Mention how participants were recruited (online vs. offline), e.g., from an open access website or from a clinic or a closed online user group (closed usergroup trial), and clarify if this was a purely web-based trial, or there were face-to-face components (as part of the intervention or for assessment). Clearly say if outcomes were self-assessed through questionnaires (as common in web-based trials). Note: In traditional offline trials, an open trial (open-label trial) is a type of clinical trial in which both the researchers and participants know which treatment is being administered. To avoid confusion, use “blinded” or “unblinded” to indicated the level of blinding instead of “open”, as “open” in web-based trials usually refers to “open access” (i.e. participants can self-enrol). (Note: Only report in the abstract what the main paper is reporting. If this information is missing from the main body of text, consider adding it)

	1	2	3	4	5	
subitem not at all important	○	○	◉	○	○	essential

Rensa markering

Does your paper address subitem 1b-iii?

Copy and paste relevant sections from the manuscript abstract (include quotes in quotation marks "like this" to indicate direct quotes from your manuscript), or elaborate on this item by providing additional information not in the ms, or briefly explain why the item is not applicable/relevant for your study

Yes partly in "Methods: A two-armed, non-blinded randomized controlled trial was conducted. Both groups received usual care; the intervention group additionally participated in a PCC intervention via telephone and an eHealth platform. The primary outcome was a composite score consisting of change in general self-efficacy and self-reported sick leave at 6-month follow-up."

1b-iv) RESULTS section in abstract must contain use data

Report number of participants enrolled/assessed in each group, the use/uptake of the intervention (e.g., attrition/adherence metrics, use over time, number of logins etc.), in addition to primary/secondary outcomes. (Note: Only report in the abstract what the main paper is reporting. If this information is missing from the main body of text, consider adding it)

	1	2	3	4	5	
subitem not at all important	○	○	◉	○	○	essential

Rensa markering

Does your paper address subitem 1b-iv?

Copy and paste relevant sections from the manuscript abstract (include quotes in quotation marks "like this" to indicate direct quotes from your manuscript), or elaborate on this item by providing additional information not in the ms, or briefly explain why the item is not applicable/relevant for your study

Yes, "Results: A total of 59 participants were randomized to the intervention (n=29) and control group (n=30)."

1b-v) CONCLUSIONS/DISCUSSION in abstract for negative trials

Conclusions/Discussions in abstract for negative trials: Discuss the primary outcome - if the trial is negative (primary outcome not changed), and the intervention was not used, discuss whether negative results are attributable to lack of uptake and discuss reasons. (Note: Only report in the abstract what the main paper is reporting. If this information is missing from the main body of text, consider adding it)

	1	2	3	4	5	
subitem not at all important	○	○	◉	○	○	essential

Rensa markering

Does your paper address subitem 1b-v?

Copy and paste relevant sections from the manuscript abstract (include quotes in quotation marks "like this" to indicate direct quotes from your manuscript), or elaborate on this item by providing additional information not in the ms, or briefly explain why the item is not applicable/relevant for your study

Not applicable; this was not a negative trial, as the primary outcome showed a statistically significant difference between groups.

INTRODUCTION

2a) In INTRODUCTION: Scientific background and explanation of rationale

2a-i) Problem and the type of system/solution

Describe the problem and the type of system/solution that is object of the study: intended as stand-alone intervention vs. incorporated in broader health care program? Intended for a particular patient population? Goals of the intervention, e.g., being more cost-effective to other interventions, replace or complement other solutions? (Note: Details about the intervention are provided in "Methods" under 5)

	1	2	3	4	5	
subitem not at all important	○	○	○	○	◉	essential

Rensa markering

Does your paper address subitem 2a-i? *

Copy and paste relevant sections from the manuscript (include quotes in quotation marks "like this" to indicate direct quotes from your manuscript), or elaborate on this item by providing additional information not in the ms, or briefly explain why the item is not applicable/relevant for your study

Yes, the problem and its intended population "Chronic pain affects 19–20% of the population in Europe and North America (1,2) and is the single greatest cause of years lived with disability (YLDs) (3). Chronic pain is associated with psychiatric comorbidity, cardiovascular diseases and cancer (4), elevated risk of suicidal ideation and suicide attempts (5) and premature mortality (6). Furthermore, chronic pain negatively impacts quality of life and daily activities, as well as bringing repercussions to the person's social and family environment (7). Chronic pain significantly impacts ability to work (8,9) leading to increased sick leave and economic burdens at both personal and societal levels (10). Work absences account for the majority of the estimated cost of chronic pain, in European countries reaching 4–10% of gross domestic product (GDP) (11,12). In 2021, the economic burden of chronic pain in the United States was estimated at 722.8 billion USD (13). Given the impact of chronic pain on workforce participation and economic stability, addressing chronic pain-related sick leave is a critical public health priority."

Object of study "Person-centred care (PCC) is a practised ethic (15) founded on the establishment of a partnership between the patient and health care professionals (HCPs), and possibly others such as family members (16,17). This partnership is based on mutual trust, understanding and shared knowledge, accomplished through genuine engagement, empathetic presence, shared decision making and integration of the patient's beliefs, values, needs and resources (17,18). Previous PCC interventions delivered remotely via telephone and an eHealth platform in a primary care context have shown improvement in self-efficacy in persons with chronic heart failure and common mental disorders (19,20)."

2a-ii) Scientific background, rationale: What is known about the (type of) system

Scientific background, rationale: What is known about the (type of) system that is the object of the study (be sure to discuss the use of similar systems for other conditions/diagnoses, if appropiate), motivation for the study, i.e. what are the reasons for and what is the context for this specific study, from which stakeholder viewpoint is the study performed, potential impact of findings [2]. Briefly justify the choice of the comparator.

	1	2	3	4	5	
subitem not at all important	○	○	○	●	○	essential

Rensa markering

Does your paper address subitem 2a-ii? *

Copy and paste relevant sections from the manuscript (include quotes in quotation marks "like this" to indicate direct quotes from your manuscript), or elaborate on this item by providing additional information not in the ms, or briefly explain why the item is not applicable/relevant for your study

Yes "Person-centred care (PCC) is a practised ethic (15) founded on the establishment of a partnership between the patient and health care professionals (HCPs), and possibly others such as family members (16,17). This partnership is based on mutual trust, understanding and shared knowledge, accomplished through genuine engagement, empathetic presence, shared decision making and integration of the patient’s beliefs, values, needs and resources (17,18). Previous PCC interventions delivered remotely via telephone and an eHealth platform in a primary care context have shown improvement in self-efficacy in persons with chronic heart failure and common mental disorders (19,20). “Self-efficacy” refers to a person’s belief in their ability to succeed in certain situations (21) and its importance in facilitating return to work has been highlighted in several studies (22–24). Self-efficacy is important in relation to chronic pain as higher self-efficacy is associated with positive treatment outcomes for physical functioning and level of disability (25), work status, and lower pain intensity (26), while lower or reduced self-efficacy is a negative predictor for return to work (27). However, little is known about the efficacy of PCC for persons on sick leave due to chronic pain. Therefore, this study aims to evaluate the efficacy of a person-centred intervention at home, consisting of a combined telephone support and eHealth platform, in persons on sick leave due to chronic pain."

2b) In INTRODUCTION: Specific objectives or hypotheses

Does your paper address CONSORT subitem 2b? *

Copy and paste relevant sections from the manuscript (include quotes in quotation marks "like this" to indicate direct quotes from your manuscript), or elaborate on this item by providing additional information not in the ms, or briefly explain why the item is not applicable/relevant for your study

Yes "this study aims to evaluate the efficacy of a person-centred intervention at home, consisting of a combined telephone support and eHealth platform, in persons on sick leave due to chronic pain." (page 3) and "open randomized controlled trial (RCT) hypothesizing that, among persons on sick leave due to chronic pain, those who receive a PCC intervention will have an improved composite score combining self-reported self-efficacy and sick leave compared with controls. Accordingly, the null hypothesis assumed no differences between the intervention and control groups at endpoint." (page 4)

METHODS

3a) Description of trial design (such as parallel, factorial) including allocation ratio

Does your paper address CONSORT subitem 3a? *

Copy and paste relevant sections from the manuscript (include quotes in quotation marks "like this" to indicate direct quotes from your manuscript), or elaborate on this item by providing additional information not in the ms, or briefly explain why the item is not applicable/relevant for your study

Yes "was a two-arm, non-blinded, open randomized controlled trial (RCT)" and "Participants were individually allocated 1:1 to either the control group or the intervention group."

3b) Important changes to methods after trial commencement (such as eligibility criteria), with reasons

Does your paper address CONSORT subitem 3b? *

Copy and paste relevant sections from the manuscript (include quotes in quotation marks "like this" to indicate direct quotes from your manuscript), or elaborate on this item by providing additional information not in the ms, or briefly explain why the item is not applicable/relevant for your study

There were no important changes to the methods after trial commencement.

3b-i) Bug fixes, Downtimes, Content Changes

Bug fixes, Downtimes, Content Changes: ehealth systems are often dynamic systems. A description of changes to methods therefore also includes important changes made on the intervention or comparator during the trial (e.g., major bug fixes or changes in the functionality or content) (5-iii) and other “unexpected events” that may have influenced study design such as staff changes, system failures/downtimes, etc. [2].

	1	2	3	4	5	
subitem not at all important	○	○	○	◉	○	essential

Rensa markering

Does your paper address subitem 3b-i?

Copy and paste relevant sections from the manuscript (include quotes in quotation marks "like this" to indicate direct quotes from your manuscript), or elaborate on this item by providing additional information not in the ms, or briefly explain why the item is not applicable/relevant for your study

No as there were no major bug fixes, functionality or content changes, system downtimes, or other unexpected events that influenced the study during the trial.

4a) Eligibility criteria for participants

Does your paper address CONSORT subitem 4a? *

Copy and paste relevant sections from the manuscript (include quotes in quotation marks "like this" to indicate direct quotes from your manuscript), or elaborate on this item by providing additional information not in the ms, or briefly explain why the item is not applicable/relevant for your study

Yes, "Inclusion criteria were: men and women aged 18–65 on sick leave due to chronic, non-malignant pain with any of the following diagnoses according to the International Statistical Classification of Diseases and Related Health Problems, 10th revision (ICD-10) (30): M25 – other joint disorders not classified elsewhere; M54 – dorsalgia; M79 – other soft tissue disorders not elsewhere classified; or R52 – pain not elsewhere classified. Exclusion criteria were: being on full-time sick leave ≥24 months; severe cognitive or physical impairment (i.e. preventing the person from using the eHealth support); severe disease other than chronic pain, with expected survival ≤12 months; present documented diagnosis of alcohol or drug abuse; participating in another, conflicting RCT; not understanding written and spoken Swedish; not having access to a digital device with internet access; and having no registered address. "

4a-i) Computer / Internet literacy

Computer / Internet literacy is often an implicit “de facto” eligibility criterion - this should be explicitly clarified.

	1	2	3	4	5	
subitem not at all important	○	○	○	◉	○	essential

Rensa markering

Does your paper address subitem 4a-i?

Copy and paste relevant sections from the manuscript (include quotes in quotation marks "like this" to indicate direct quotes from your manuscript), or elaborate on this item by providing additional information not in the ms, or briefly explain why the item is not applicable/relevant for your study

Yes "not having access to a digital device with internet access".
Basic computer/Internet literacy was implicitly required to use the intervention; eligibility was therefore limited to participants with access to and the ability to use a digital device.

4a-ii) Open vs. closed, web-based vs. face-to-face assessments:

Open vs. closed, web-based vs. face-to-face assessments: Mention how participants were recruited (online vs. offline), e.g., from an open access website or from a clinic, and clarify if this was a purely web-based trial, or there were face-to-face components (as part of the intervention or for assessment), i.e., to what degree got the study team to know the participant. In online-only trials, clarify if participants were quasi-anonymous and whether having multiple identities was possible or whether technical or logistical measures (e.g., cookies, email confirmation, phone calls) were used to detect/prevent these.

	1	2	3	4	5	
subitem not at all important	○	○	○	◉	○	essential

Rensa markering

Does your paper address subitem 4a-ii? *

Copy and paste relevant sections from the manuscript (include quotes in quotation marks "like this" to indicate direct quotes from your manuscript), or elaborate on this item by providing additional information not in the ms, or briefly explain why the item is not applicable/relevant for your study

Yes "Participants were recruited from ten primary health care centres in a socioeconomically diverse area of Gothenburg, Sweden" and "Recruitment took place between August 2021 and June 2023. Medical records from the participating health care centres were continuously screened for eligible participants by a designated HCP. Letters containing information about the study, contact details and a note indicating that the person would be contacted by telephone for further information about the study were sent to eligible participants. Further information about the study was provided over the phone, and those interested in participating were sent written informed consent forms via post together with a pre-paid return envelope. After return of a signed consent form, the participants were randomized to either the intervention or the control group by the same designated HCP. Randomization was performed using a web-based randomization software; dSharp Randomization (dSharp Consulting & Statistiska Konsultgruppen, Gothenburg, Sweden) with stratification for age and sex. Participants were informed about their randomized allocation by telephone. "

4a-iii) Information giving during recruitment

Information given during recruitment. Specify how participants were briefed for recruitment and in the informed consent procedures (e.g., publish the informed consent documentation as appendix, see also item X26), as this information may have an effect on user self-selection, user expectation and may also bias results.

	1	2	3	4	5	
subitem not at all important	○	○	◉	○	○	essential

Rensa markering

Does your paper address subitem 4a-iii?

Copy and paste relevant sections from the manuscript (include quotes in quotation marks "like this" to indicate direct quotes from your manuscript), or elaborate on this item by providing additional information not in the ms, or briefly explain why the item is not applicable/relevant for your study

"Letters containing information about the study, contact details and a note indicating that the person would be contacted by telephone for further information about the study were sent to eligible participants. Further information about the study was provided over the phone, and those interested in participating were sent written informed consent forms via post together with a pre-paid return envelope. After return of a signed consent form, the participants were randomized to either the intervention or the control group by the same designated HCP."

4b) Settings and locations where the data were collected

Does your paper address CONSORT subitem 4b? *

Copy and paste relevant sections from the manuscript (include quotes in quotation marks "like this" to indicate direct quotes from your manuscript), or elaborate on this item by providing additional information not in the ms, or briefly explain why the item is not applicable/relevant for your study

Yes "Participants were recruited from ten primary health care centres in a socioeconomically diverse area of Gothenburg" and "The HCPs carrying out the intervention operated from a research unit at the hospital and were not involved in the participants' usual care or connected to the primary health care centres from which the participants were recruited. "

4b-i) Report if outcomes were (self-)assessed through online questionnaires

Clearly report if outcomes were (self-)assessed through online questionnaires (as common in web-based trials) or otherwise.

	1	2	3	4	5	
subitem not at all important	○	○	●	○	○	essential

Rensa markering

Does your paper address subitem 4b-i? *

Copy and paste relevant sections from the manuscript (include quotes in quotation marks "like this" to indicate direct quotes from your manuscript), or elaborate on this item by providing additional information not in the ms, or briefly explain why the item is not applicable/relevant for your study

Yes, outcomes were self-assessed but through mail/paper questionnaires see "Participants answered questionnaires by post at baseline and at 3 and 6 months after inclusion"

4b-ii) Report how institutional affiliations are displayed

Report how institutional affiliations are displayed to potential participants [on ehealth media], as affiliations with prestigious hospitals or universities may affect volunteer rates, use, and reactions with regards to an intervention.(Not a required item – describe only if this may bias results)

	1	2	3	4	5	
subitem not at all important	●	○	○	○	○	essential

Rensa markering

Does your paper address subitem 4b-ii?

Copy and paste relevant sections from the manuscript (include quotes in quotation marks "like this" to indicate direct quotes from your manuscript), or elaborate on this item by providing additional information not in the ms, or briefly explain why the item is not applicable/relevant for your study

Ditt svar

5) The interventions for each group with sufficient details to allow replication, including how and when they were actually administered

5-i) Mention names, credential, affiliations of the developers, sponsors, and owners

Mention names, credential, affiliations of the developers, sponsors, and owners [6] (if authors/evaluators are owners or developer of the software, this needs to be declared in a “Conflict of interest” section or mentioned elsewhere in the manuscript).

	1	2	3	4	5	
subitem not at all important	○	◉	○	○	○	essential

Rensa markering

Does your paper address subitem 5-i?

Copy and paste relevant sections from the manuscript (include quotes in quotation marks "like this" to indicate direct quotes from your manuscript), or elaborate on this item by providing additional information not in the ms, or briefly explain why the item is not applicable/relevant for your study

Yes "The eHealth platform was developed at the University of Gothenburg through collaboration among information technology (IT) developers, HCPs, researchers, and research partners"

5-ii) Describe the history/development process

Describe the history/development process of the application and previous formative evaluations (e.g., focus groups, usability testing), as these will have an impact on adoption/use rates and help with interpreting results.

	1	2	3	4	5	
subitem not at all important	○	○	○	◉	○	essential

Rensa markering

Does your paper address subitem 5-ii?

Copy and paste relevant sections from the manuscript (include quotes in quotation marks "like this" to indicate direct quotes from your manuscript), or elaborate on this item by providing additional information not in the ms, or briefly explain why the item is not applicable/relevant for your study

Yes "Additionally, several other research partners have been actively involved in the design phase of the projects preceding our trial, in which the eHealth platform used in our intervention was developed. The eHealth platform was developed at the University of Gothenburg through collaboration among information technology (IT) developers, HCPs, researchers, and research partners, where the research partners were active in its development through a participatory process, offering insights on its viability and later modification of the platform content to accommodate different target populations (40)"

5-iii) Revisions and updating

Revisions and updating. Clearly mention the date and/or version number of the application/intervention (and comparator, if applicable) evaluated, or describe whether the intervention underwent major changes during the evaluation process, or whether the development and/or content was “frozen” during the trial. Describe dynamic components such as news feeds or changing content which may have an impact on the replicability of the intervention (for unexpected events see item 3b).

	1	2	3	4	5	
subitem not at all important	○	○	○	◉	○	essential

Rensa markering

Does your paper address subitem 5-iii?

Copy and paste relevant sections from the manuscript (include quotes in quotation marks "like this" to indicate direct quotes from your manuscript), or elaborate on this item by providing additional information not in the ms, or briefly explain why the item is not applicable/relevant for your study

No, as the intervention did not undergo any major revisions during the evaluation period. Only routine maintenance occurred, with no changes to content, functionality, or user experience.

5-iv) Quality assurance methods

Provide information on quality assurance methods to ensure accuracy and quality of information provided [1], if applicable.

	1	2	3	4	5	
subitem not at all important	○	◉	○	○	○	essential

Rensa markering

Does your paper address subitem 5-iv?

Copy and paste relevant sections from the manuscript (include quotes in quotation marks "like this" to indicate direct quotes from your manuscript), or elaborate on this item by providing additional information not in the ms, or briefly explain why the item is not applicable/relevant for your study

Not applicable; no formal quality assurance methods were used.

5-v) Ensure replicability by publishing the source code, and/or providing screenshots/screen-capture video, and/or providing flowcharts of the algorithms used

Ensure replicability by publishing the source code, and/or providing screenshots/screen-capture video, and/or providing flowcharts of the algorithms used. Replicability (i.e., other researchers should in principle be able to replicate the study) is a hallmark of scientific reporting.

	1	2	3	4	5	
subitem not at all important	○	○	○	◉	○	essential

Rensa markering

Does your paper address subitem 5-v?

Copy and paste relevant sections from the manuscript (include quotes in quotation marks "like this" to indicate direct quotes from your manuscript), or elaborate on this item by providing additional information not in the ms, or briefly explain why the item is not applicable/relevant for your study

Screenshots of the eHealth website used will be uploaded as figures in the submission process.

5-vi) Digital preservation

Digital preservation: Provide the URL of the application, but as the intervention is likely to change or disappear over the course of the years; also make sure the intervention is archived (Internet Archive, webcitation.org, and/or publishing the source code or screenshots/videos alongside the article). As pages behind login screens cannot be archived, consider creating demo pages which are accessible without login.

	1	2	3	4	5	
subitem not at all important	○	○	◉	○	○	essential

Rensa markering

Does your paper address subitem 5-vi?

Copy and paste relevant sections from the manuscript (include quotes in quotation marks "like this" to indicate direct quotes from your manuscript), or elaborate on this item by providing additional information not in the ms, or briefly explain why the item is not applicable/relevant for your study

The intervention is accessible via a secure login at [https://minhalsa.smarta.it.gu.se/forskningsperson/home] and therefore cannot be archived using public web archiving services.

5-vii) Access

Access: Describe how participants accessed the application, in what setting/context, if they had to pay (or were paid) or not, whether they had to be a member of specific group. If known, describe how participants obtained “access to the platform and Internet” [1]. To ensure access for editors/reviewers/readers, consider to provide a “backdoor” login account or demo mode for reviewers/readers to explore the application (also important for archiving purposes, see vi).

	1	2	3	4	5	
subitem not at all important	○	○	○	◉	○	essential

Rensa markering

Does your paper address subitem 5-vii? *

Copy and paste relevant sections from the manuscript (include quotes in quotation marks "like this" to indicate direct quotes from your manuscript), or elaborate on this item by providing additional information not in the ms, or briefly explain why the item is not applicable/relevant for your study

"The health plan was written down (by the HCP or the participant, based on the participant’s preferences) and uploaded to the digital eHealth platform called MyHealth. Using a personal login on a computer or smartphone, the participants were able to access and add or revise a health plan at any time on the eHealth platform. "

5-viii) Mode of delivery, features/functionalities/components of the intervention and comparator, and the theoretical framework

Describe mode of delivery, features/functionalities/components of the intervention and comparator, and the theoretical framework [6] used to design them (instructional strategy [1], behaviour change techniques, persuasive features, etc., see e.g., [7, 8] for terminology). This includes an in-depth description of the content (including where it is coming from and who developed it) [1]," whether [and how] it is tailored to individual circumstances and allows users to track their progress and receive feedback" [6]. This also includes a description of communication delivery channels and – if computer-mediated communication is a component – whether communication was synchronous or asynchronous [6]. It also includes information on presentation strategies [1], including page design principles, average amount of text on pages, presence of hyperlinks to other resources, etc. [1].

	1	2	3	4	5	
subitem not at all important	○	○	○	◉	○	essential

Rensa markering

Does your paper address subitem 5-viii? *

Copy and paste relevant sections from the manuscript (include quotes in quotation marks "like this" to indicate direct quotes from your manuscript), or elaborate on this item by providing additional information not in the ms, or briefly explain why the item is not applicable/relevant for your study

Yes "The intervention group, in addition to usual care, participated in a PCC intervention consisting of two parts; telephone calls with HCPs combined with access to an eHealth platform, during a 6-month period. The HCPs carrying out the intervention were three registered nurses and a registered physiotherapist. One had extensive previous experience in providing PCC through telephone and eHealth platforms, while the others received initial training prior to the study, consisting of coursework in PCC philosophy equivalent to 7.5 ECTS (European Credit Transfer and Accumulation System). Thereafter, all HCPs providing the intervention continued training as a group through recurring educational seminars and mentoring sessions in practicing PCC. These were held approximately once a month throughout the intervention, led by senior researchers experienced in PCC. The HCPs carrying out the intervention operated from a research unit at the hospital and were not involved in the participants' usual care or connected to the primary health care centres from which the participants were recruited.
To ensure there is a partnership in PCC the intervention followed the three routines (initiating, working, and safeguarding the partnership) described by Ekman et al. (17). Initiating the partnership means encouraging and actively listening to the participant's narrative about their pain, life situation, needs, resources and preferences. Working the partnership involves sharing information, shared deliberation and decision making to reach a common understanding and common goals. Safeguarding the partnership includes documenting the patient's narrative, the mutually agreed health plan and actions to achieve the shared short-term and long-term goals.
Telephone calls
The participants booked at least one initial phone call with the HCP and then scheduled follow-up calls as needed during the 6-month intervention period. During the calls, the participants were encouraged to narrate their experiences by the HCPs, who asked open-ended questions about the participants' current situation, and listened and asked follow-up questions. During the conversations the participants' resources were identified, both long-term and short-term goals were formulated and a jointly agreed health plan was created. The health plan contained information relevant to the participant, such as a summary of the participant's situation, their resources, and goals, and the support they needed to achieve those goals. The health plan was continuously updated and followed-up throughout the intervention, usually in connection with a scheduled call. Toward the end of each call, a subsequent call was jointly scheduled with the participant, based on the participant's wishes and needs, typically at intervals of 2–4 weeks.
eHealth platform
The health plan was written down (by the HCP or the participant, based on the participant's preferences) and uploaded to the digital eHealth platform called MyHealth. Using a personal login on a computer or smartphone, the participants were able to access and add or revise a health plan at any time on the eHealth platform. They could use the platform to send messages to the HCPs, see the date and time for their next agreed-upon phone call, invite others (their social networks and/or regular health care contacts) to the platform and rate their sleep, activities, pain and anxiety on a 5-point Likert scale. Answers to the questions created a graph that enabled both participants and HCPs to follow a trend. These ratings on the eHealth platform were used to facilitate and tailor conversations during the calls with HCPs; they were not extracted or used in the final statistical analyses."

5-ix) Describe use parameters

Describe use parameters (e.g., intended “doses” and optimal timing for use). Clarify what instructions or recommendations were given to the user, e.g., regarding timing, frequency, heaviness of use, if any, or was the intervention used ad libitum.

	1	2	3	4	5	
subitem not at all important	○	○	○	◉	○	essential

Rensa markering

Does your paper address subitem 5-ix?

Copy and paste relevant sections from the manuscript (include quotes in quotation marks "like this" to indicate direct quotes from your manuscript), or elaborate on this item by providing additional information not in the ms, or briefly explain why the item is not applicable/relevant for your study

Yes "The participants booked at least one initial phone call with the HCP and then scheduled follow-up calls as needed during the 6-month intervention period." and "The health plan was continuously updated and followed-up throughout the intervention, usually in connection with a scheduled call. Toward the end of each call, a subsequent call was jointly scheduled with the participant, based on the participant’s wishes and needs, typically at intervals of 2–4 weeks."

5-x) Clarify the level of human involvement

Clarify the level of human involvement (care providers or health professionals, also technical assistance) in the e-intervention or as co-intervention (detail number and expertise of professionals involved, if any, as well as “type of assistance offered, the timing and frequency of the support, how it is initiated, and the medium by which the assistance is delivered”. It may be necessary to distinguish between the level of human involvement required for the trial, and the level of human involvement required for a routine application outside of a RCT setting (discuss under item 21 – generalizability).

	1	2	3	4	5	
subitem not at all important	○	○	○	◉	○	essential

Rensa markering

Does your paper address subitem 5-x?

Copy and paste relevant sections from the manuscript (include quotes in quotation marks "like this" to indicate direct quotes from your manuscript), or elaborate on this item by providing additional information not in the ms, or briefly explain why the item is not applicable/relevant for your study

Yes ". The HCPs carrying out the intervention were three registered nurses and a registered physiotherapist. One had extensive previous experience in providing PCC through telephone and eHealth platforms, while the others received initial training prior to the study, consisting of coursework in PCC philosophy equivalent to 7.5 ECTS (European Credit Transfer and Accumulation System). Thereafter, all HCPs providing the intervention continued training as a group through recurring educational seminars and mentoring sessions in practicing PCC. These were held approximately once a month throughout the intervention, led by senior researchers experienced in PCC."

"The participants booked at least one initial phone call with the HCP and then scheduled follow-up calls as needed during the 6-month intervention period. During the calls, the participants were encouraged to narrate their experiences by the HCPs, who asked open-ended questions about the participants' current situation, and listened and asked follow-up questions. During the conversations the participants' resources were identified, both long-term and short-term goals were formulated and a jointly agreed health plan was created. The health plan contained information relevant to the participant, such as a summary of the participant's situation, their resources, and goals, and the support they needed to achieve those goals. The health plan was continuously updated and followed-up throughout the intervention, usually in connection with a scheduled call. Toward the end of each call, a subsequent call was jointly scheduled with the participant, based on the participant's wishes and needs, typically at intervals of 2–4 weeks."

"The health plan was written down (by the HCP or the participant, based on the participant's preferences) and uploaded to the digital eHealth platform called MyHealth. Using a personal login on a computer or smartphone, the participants were able to access and add or revise a health plan at any time on the eHealth platform. They could use the platform to send messages to the HCPs, see the date and time for their next agreed-upon phone call, invite others (their social networks and/or regular health care contacts) to the platform and rate their sleep, activities, pain and anxiety on a 5-point Likert scale. "

5-xi) Report any prompts/reminders used

Report any prompts/reminders used: Clarify if there were prompts (letters, emails, phone calls, SMS) to use the application, what triggered them, frequency etc. It may be necessary to distinguish between the level of prompts/reminders required for the trial, and the level of prompts/reminders for a routine application outside of a RCT setting (discuss under item 21 – generalizability).

	1	2	3	4	5	
subitem not at all important	○	○	○	◉	○	essential

Rensa markering

Does your paper address subitem 5-xi? *

Copy and paste relevant sections from the manuscript (include quotes in quotation marks "like this" to indicate direct quotes from your manuscript), or elaborate on this item by providing additional information not in the ms, or briefly explain why the item is not applicable/relevant for your study

No prompts or reminders (e.g., emails, SMS, phone calls) were used to encourage application use during the trial. The application and phone calls were scheduled and used based on the need of the patient and a joint planning with the HCP. However the participants could contact the HCP's through the platform earlier if needed.

5-xii) Describe any co-interventions (incl. training/support)

Describe any co-interventions (incl. training/support): Clearly state any interventions that are provided in addition to the targeted eHealth intervention, as ehealth intervention may not be designed as stand-alone intervention. This includes training sessions and support [1]. It may be necessary to distinguish between the level of training required for the trial, and the level of training for a routine application outside of a RCT setting (discuss under item 21 – generalizability.

	1	2	3	4	5	
subitem not at all important	○	○	○	◉	○	essential

Rensa markering

Does your paper address subitem 5-xii? *

Copy and paste relevant sections from the manuscript (include quotes in quotation marks "like this" to indicate direct quotes from your manuscript), or elaborate on this item by providing additional information not in the ms, or briefly explain why the item is not applicable/relevant for your study

In addition to the eHealth intervention, structured telephone calls with HCP see "The intervention group, in addition to usual care, participated in a PCC intervention consisting of two parts; telephone calls with HCPs combined with access to an eHealth platform, during a 6-month period." and "The participants booked at least one initial phone call with the HCP and then scheduled follow-up calls as needed during the 6-month intervention period. During the calls, the participants were encouraged to narrate their experiences by the HCPs, who asked open-ended questions about the participants' current situation, and listened and asked follow-up questions. During the conversations the participants' resources were identified, both long-term and short-term goals were formulated and a jointly agreed health plan was created. The health plan contained information relevant to the participant, such as a summary of the participant's situation, their resources, and goals, and the support they needed to achieve those goals. The health plan was continuously updated and followed-up throughout the intervention, usually in connection with a scheduled call. Toward the end of each call, a subsequent call was jointly scheduled with the participant, based on the participant's wishes and needs, typically at intervals of 2–4 weeks."

6a) Completely defined pre-specified primary and secondary outcome measures, including how and when they were assessed

Does your paper address CONSORT subitem 6a? *

Copy and paste relevant sections from the manuscript (include quotes in quotation marks "like this" to indicate direct quotes from your manuscript), or elaborate on this item by providing additional information not in the ms, or briefly explain why the item is not applicable/relevant for your study

Yes, "At inclusion, data on sex, age, diagnosis, comorbidities and medications were collected from the medical records by a designated HCP. Participants answered questionnaires by post at baseline and at 3 and 6 months after inclusion (endpoint). At baseline, self-reported data on country of birth, level of education, occupation and social status were collected, while primary outcome data on general self-efficacy and sick leave ratio were collected on all three occasions. Self-efficacy was assessed using the Swedish version (33) of the General Self-Efficacy scale (GSE) (34). The GSE scale is a ten-item questionnaire used to assess a person's belief in their ability to handle problems and associated difficulties (Löve et al., 2012). The items in the GSE scale consist of statements answered on a Likert scale of 1–4, where 1 = "not at all true", 2 = "hardly true", 3 = "moderately true" and 4 = "exactly true". The score adds up to between 10 and 40, where higher scores correspond to higher self-efficacy (34). The scale has shown high internal consistency (Cronbach's alpha = 0.91) when explored in relation to work capacity and sick leave in a randomized general population (33). Sick leave was measured based on the participants' self-reported current percentage of sick leave in relation to full-time work, on a scale of 0–100. Participants were instructed to report their current level of continuous sick leave, rather than isolated sick days due to minor illnesses (e.g., colds). "

"The primary outcome consisted of a composite of changes in self-reported GSE and sick leave level at endpoint (6-month follow-up). A composite score merges two or more relevant variables into a single measure (35). Initially developed within efficacy trials in cardiology (36), it is now a commonly applied approach in health research and serves as a valuable tool for evaluation of clinical trials as they provide statistical advantages including reduced sample size requirement and reduced follow-up period in trials (37). The composite score used in this RCT was defined a priori, and to provide transparency in reporting the results of each individual variable was presented separately (37).
The participants were classified as improved, deteriorated or unchanged. Participants were classified as improved if their GSE score had increased by ≥5 points and if they had a reduced sick leave level compared with baseline. They were classified as deteriorated if their GSE score had decreased by ≥5 points and/or they had increased sick leave compared with baseline. Participants who did not fulfil the criteria for either improvement or deterioration were classified as unchanged. Previous research has considered a 5-point change in GSE to be a threshold for minimal important change (38,39). Sick leave level and GSE as a composite score have been previously used in other RCTs evaluating PCC (20)."

Also see registration in ClinicalTrials.gov identifier: NCT04706195, submitted 2021-01-09 and Multimedia Appendix 3: Statistical analysis plan

6a-i) Online questionnaires: describe if they were validated for online use and apply CHERRIES items to describe how the questionnaires were designed/deployed

If outcomes were obtained through online questionnaires, describe if they were validated for online use and apply CHERRIES items to describe how the questionnaires were designed/deployed [9].

	1	2	3	4	5	
subitem not at all important	○	○	○	○	◉	essential

Rensa markering

Does your paper address subitem 6a-i?

Copy and paste relevant sections from manuscript text

Not applicable; outcomes were not obtained using online questionnaires.

6a-ii) Describe whether and how “use” (including intensity of use/dosage) was defined/measured/monitored

Describe whether and how “use” (including intensity of use/dosage) was defined/measured/monitored (logins, logfile analysis, etc.). Use/adoption metrics are important process outcomes that should be reported in any ehealth trial.

	1	2	3	4	5	
subitem not at all important	○	○	◉	○	○	essential

Rensa markering

Does your paper address subitem 6a-ii?

Copy and paste relevant sections from manuscript text

The participants used the eHealth tool and telephone support as much as needed. The final number of health plans and telephone calls are reported in the results.

6a-iii) Describe whether, how, and when qualitative feedback from participants was obtained

Describe whether, how, and when qualitative feedback from participants was obtained (e.g., through emails, feedback forms, interviews, focus groups).

	1	2	3	4	5	
subitem not at all important	○	○	◉	○	○	essential

Rensa markering

Does your paper address subitem 6a-iii?

Copy and paste relevant sections from manuscript text

No qualitative feedback from participants was obtained during the study.

6b) Any changes to trial outcomes after the trial commenced, with reasons

Does your paper address CONSORT subitem 6b? *

Copy and paste relevant sections from the manuscript (include quotes in quotation marks "like this" to indicate direct quotes from your manuscript), or elaborate on this item by providing additional information not in the ms, or briefly explain why the item is not applicable/relevant for your study

No changes to the prespecified trial outcomes were made after trial commencement.

7a) How sample size was determined

NPT: When applicable, details of whether and how the clustering by care provides or centers was addressed

7a-i) Describe whether and how expected attrition was taken into account when calculating the sample size

Describe whether and how expected attrition was taken into account when calculating the sample size.

	1	2	3	4	5	
subitem not at all important	○	○	○	◉	○	essential

Rensa markering

Does your paper address subitem 7a-i?

Copy and paste relevant sections from manuscript title (include quotes in quotation marks "like this" to indicate direct quotes from your manuscript), or elaborate on this item by providing additional information not in the ms, or briefly explain why the item is not applicable/relevant for your study

"Assuming an improvement in the composite score of 20% in the control group and 40% in the intervention group, an alpha level of 0.05, power of 80%, using a two-sided Fisher's exact test, 91 patients per group were calculated to be needed to be included in the study. " Hence, potential attrition was considered, but no formal adjustment was made to the sample size calculation.

7b) When applicable, explanation of any interim analyses and stopping guidelines

Does your paper address CONSORT subitem 7b? *

Copy and paste relevant sections from the manuscript (include quotes in quotation marks "like this" to indicate direct quotes from your manuscript), or elaborate on this item by providing additional information not in the ms, or briefly explain why the item is not applicable/relevant for your study

Yes "No interim analyses were planned a priori or conducted during the course of the randomized controlled trial. All analyses were performed after study completion."

and "Because the inclusion rate was slower than expected, the research group deemed it infeasible to continue recruitment until desired statistical power was achieved. Thus, inclusion was terminated at 22 months, in June 2023"

8a) Method used to generate the random allocation sequence
NPT: When applicable, how care providers were allocated to each trial group

Does your paper address CONSORT subitem 8a? *

Copy and paste relevant sections from the manuscript (include quotes in quotation marks "like this" to indicate direct quotes from your manuscript), or elaborate on this item by providing additional information not in the ms, or briefly explain why the item is not applicable/relevant for your study

"Participants were individually allocated 1:1 to either the control group or the intervention group" and "After return of a signed consent form, the participants were randomized to either the intervention or the control group by the same designated HCP. Randomization was performed using a web-based randomization software; dSharp Randomization (dSharp Consulting & Statistiska Konsultgruppen, Gothenburg, Sweden) with stratification for age and sex. Participants were informed about their randomized allocation by telephone. A flowchart of enrolment, allocation, follow-up and analysis is presented in Figure 1. "

8b) Type of randomisation; details of any restriction (such as blocking and block size)

Does your paper address CONSORT subitem 8b? *

Copy and paste relevant sections from the manuscript (include quotes in quotation marks "like this" to indicate direct quotes from your manuscript), or elaborate on this item by providing additional information not in the ms, or briefly explain why the item is not applicable/relevant for your study

Yes, stratification for age and sex see "Randomization was performed using a web-based randomization software; dSharp Randomization (dSharp Consulting & Statistiska Konsultgruppen, Gothenburg, Sweden) with stratification for age and sex"
No blocking was used.

9) Mechanism used to implement the random allocation sequence (such as sequentially numbered containers), describing any steps taken to conceal the sequence until interventions were assigned

Does your paper address CONSORT subitem 9? *

Copy and paste relevant sections from the manuscript (include quotes in quotation marks "like this" to indicate direct quotes from your manuscript), or elaborate on this item by providing additional information not in the ms, or briefly explain why the item is not applicable/relevant for your study

Yes, it is implicit in the web-randomization software, "After return of a signed consent form, the participants were randomized to either the intervention or the control group by the same designated HCP. Randomization was performed using a web-based randomization software; dSharp Randomization (dSharp Consulting & Statistiska Konsultgruppen, Gothenburg, Sweden) with stratification for age and sex"

10) Who generated the random allocation sequence, who enrolled participants, and who assigned participants to interventions

Does your paper address CONSORT subitem 10? *

Copy and paste relevant sections from the manuscript (include quotes in quotation marks "like this" to indicate direct quotes from your manuscript), or elaborate on this item by providing additional information not in the ms, or briefly explain why the item is not applicable/relevant for your study

Yes, see "Recruitment took place between August 2021 and June 2023. Medical records from the participating health care centres were continuously screened for eligible participants by a designated HCP. Letters containing information about the study, contact details and a note indicating that the person would be contacted by telephone for further information about the study were sent to eligible participants. Further information about the study was provided over the phone, and those interested in participating were sent written informed consent forms via post together with a pre-paid return envelope. After return of a signed consent form, the participants were randomized to either the intervention or the control group by the same designated HCP. Randomization was performed using a web-based randomization software; dSharp Randomization (dSharp Consulting & Statistiska Konsultgruppen, Gothenburg, Sweden) with stratification for age and sex. Participants were informed about their randomized allocation by telephone. A flowchart of enrolment, allocation, follow-up and analysis is presented in Figure 1."

11a) If done, who was blinded after assignment to interventions (for example, participants, care providers, those assessing outcomes) and how

NPT: Whether or not administering co-interventions were blinded to group assignment

11a-i) Specify who was blinded, and who wasn’t

Specify who was blinded, and who wasn’t. Usually, in web-based trials it is not possible to blind the participants [1, 3] (this should be clearly acknowledged), but it may be possible to blind outcome assessors, those doing data analysis or those administering co-interventions (if any).

	1	2	3	4	5	
subitem not at all important	○	○	○	○	◉	essential

Rensa markering

Does your paper address subitem 11a-i? *

Copy and paste relevant sections from the manuscript (include quotes in quotation marks "like this" to indicate direct quotes from your manuscript), or elaborate on this item by providing additional information not in the ms, or briefly explain why the item is not applicable/relevant for your study

Due to the nature of the intervention, participants, care providers, and outcome assessors were not blinded to group assignment. Described in "The Early Accessible PErson-centred Rehabilitation for patients with chronic Pain (EAPER-P) trial was a two-arm, non-blinded, open randomized controlled trial (RCT) hypothesizing that, among persons on sick leave due to chronic pain, those who receive a PCC intervention will have an improved composite score combining self-reported self-efficacy and sick leave compared with controls"

However, the statistical consultants conducting the analyses were blinded to participant identity and had access only to pseudonymized datasets.

11a-ii) Discuss e.g., whether participants knew which intervention was the “intervention of interest” and which one was the “comparator”

Informed consent procedures (4a-ii) can create biases and certain expectations - discuss e.g., whether participants knew which intervention was the “intervention of interest” and which one was the “comparator”.

	1	2	3	4	5	
subitem not at all important	○	○	○	○	◉	essential

Rensa markering

Does your paper address subitem 11a-ii?

Copy and paste relevant sections from the manuscript (include quotes in quotation marks "like this" to indicate direct quotes from your manuscript), or elaborate on this item by providing additional information not in the ms, or briefly explain why the item is not applicable/relevant for your study

Yes, "Participants were informed about their randomized allocation by telephone." Thus they had knowledge if whether they were in th control or intervention group.

11b) If relevant, description of the similarity of interventions

(this item is usually not relevant for ehealth trials as it refers to similarity of a placebo or sham intervention to a active medication/intervention)

Does your paper address CONSORT subitem 11b? *

Copy and paste relevant sections from the manuscript (include quotes in quotation marks "like this" to indicate direct quotes from your manuscript), or elaborate on this item by providing additional information not in the ms, or briefly explain why the item is not applicable/relevant for your study

Not applicable; no placebo or sham intervention was used, and similarity of interventions is therefore not relevant.

12a) Statistical methods used to compare groups for primary and secondary outcomes

NPT: When applicable, details of whether and how the clustering by care providers or centers was addressed

Does your paper address CONSORT subitem 12a? *

Copy and paste relevant sections from the manuscript (include quotes in quotation marks "like this" to indicate direct quotes from your manuscript), or elaborate on this item by providing additional information not in the ms, or briefly explain why the item is not applicable/relevant for your study

Yes, "The primary outcome consisted of a composite of changes in self-reported GSE and sick leave level at endpoint (6-month follow-up). A composite score merges two or more relevant variables into a single measure (35). Initially developed within efficacy trials in cardiology (36), it is now a commonly applied approach in health research and serves as a valuable tool for evaluation of clinical trials as they provide statistical advantages including reduced sample size requirement and reduced follow-up period in trials (37). The composite score used in this RCT was defined a priori, and to provide transparency in reporting the results of each individual variable was presented separately (37).
The participants were classified as improved, deteriorated or unchanged. Participants were classified as improved if their GSE score had increased by ≥5 points and if they had a reduced sick leave level compared with baseline. They were classified as deteriorated if their GSE score had decreased by ≥5 points and/or they had increased sick leave compared with baseline. Participants who did not fulfil the criteria for either improvement or deterioration were classified as unchanged. Previous research has considered a 5-point change in GSE to be a threshold for minimal important change (38,39). Sick leave level and GSE as a composite score have been previously used in other RCTs evaluating PCC (20)."

and in "Background characteristics were calculated using descriptive and inferential statistics. Fisher's exact test was used for dichotomous variables, Mann-Whitney U-test for continuous variables and chi-square test for non-ordered categorical variables. The primary outcome was analysed using Mantel-Haenszel chi-square trend test. Mixed models for repeated measures (MMRM) were used, with an unstructured covariance pattern, for the outcome change from baseline to follow-up and adjusted for the baseline value of the outcome as a covariate in the model. For sick leave, the data were not normally distributed and therefore robust sandwich estimators were used to ensure valid inference. Least square (LS) means are model-estimated marginal means for each group at the mean baseline value of the outcome (GSE or sick leave).
As there were no major protocol violations, only intention-to-treat (ITT) analysis was performed. Missing outcome data from the composite score (GSE and sick leave level) at 3- and 6-month follow-up were imputed using last observation carried forward (LOCF). The significance level was set at p<0.05 (two-sided) and 95% confidence intervals (CIs) were reported. The statistical analysis plan (SAP) is available as supplementary material provided in Multimedia Appendix 3. SAS statistical software, version 9.4 (SAS Institute Inc., Cary, NC, USA), and IBM SPSS Statistics, version 29 (IBM, Armonk, NY, USA), were used for the calculations."

12a-i) Imputation techniques to deal with attrition / missing values

Imputation techniques to deal with attrition / missing values: Not all participants will use the intervention/comparator as intended and attrition is typically high in ehealth trials. Specify how participants who did not use the application or dropped out from the trial were treated in the statistical analysis (a complete case analysis is strongly discouraged, and simple imputation techniques such as LOCF may also be problematic [4]).

	1	2	3	4	5	
subitem not at all important	○	○	○	○	◉	essential

Rensa markering

Does your paper address subitem 12a-i? *

Copy and paste relevant sections from the manuscript (include quotes in quotation marks "like this" to indicate direct quotes from your manuscript), or elaborate on this item by providing additional information not in the ms, or briefly explain why the item is not applicable/relevant for your study

Yes. There were no dropouts during the six month trial. "As there were no major protocol violations, only intention-to-treat (ITT) analysis was performed"

On the statistical consultant's recommendation LOCF was used. "Missing outcome data from the composite score (GSE and sick leave level) at 3- and 6-month follow-up were imputed using last observation carried forward (LOCF)."

Additionally, sensitivity analyses (on non-imputed data) were performed, and is found in the Multimedia Appendices.

12b) Methods for additional analyses, such as subgroup analyses and adjusted analyses

Does your paper address CONSORT subitem 12b? *

Copy and paste relevant sections from the manuscript (include quotes in quotation marks "like this" to indicate direct quotes from your manuscript), or elaborate on this item by providing additional information not in the ms, or briefly explain why the item is not applicable/relevant for your study

No additional subgroup or adjusted analyses were performed.

X26) REB/IRB Approval and Ethical Considerations [recommended as subheading under "Methods"] (not a CONSORT item)

X26-i) Comment on ethics committee approval

	1	2	3	4	5	
subitem not at all important	○	○	○	○	◉	essential

Rensa markering

Does your paper address subitem X26-i?

Copy and paste relevant sections from the manuscript (include quotes in quotation marks "like this" to indicate direct quotes from your manuscript), or elaborate on this item by providing additional information not in the ms, or briefly explain why the item is not applicable/relevant for your study

Yes, as a subheading under "methods". See "Ethical considerations: The study adheres to the Declaration of Helsinki (41). It has been approved by the Swedish Ethical Review Authority (Reg No. 2020-02491, amendments 2020-06936 and 2021-02753). Written and verbal informed consent was obtained from each participant before participation in the study. Participant confidentiality was ensured by pseudonymisation as well as storing collected data and identifiers with restricted access. No compensation was provided for participation in this study."

x26-ii) Outline informed consent procedures

Outline informed consent procedures e.g., if consent was obtained offline or online (how? Checkbox, etc.?), and what information was provided (see 4a-ii). See [6] for some items to be included in informed consent documents.

	1	2	3	4	5	
subitem not at all important	○	○	○	◉	○	essential

Rensa markering

Does your paper address subitem X26-ii?

Copy and paste relevant sections from the manuscript (include quotes in quotation marks "like this" to indicate direct quotes from your manuscript), or elaborate on this item by providing additional information not in the ms, or briefly explain why the item is not applicable/relevant for your study

Yes, "Medical records from the participating health care centres were continuously screened for eligible participants by a designated HCP. Letters containing information about the study, contact details and a note indicating that the person would be contacted by telephone for further information about the study were sent to eligible participants. Further information about the study was provided over the phone, and those interested in participating were sent written informed consent forms via post together with a pre-paid return envelope. After return of a signed consent form, the participants were randomized to either the intervention or the control group by the same designated HCP. "

X26-iii) Safety and security procedures

Safety and security procedures, incl. privacy considerations, and any steps taken to reduce the likelihood or detection of harm (e.g., education and training, availability of a hotline)

	1	2	3	4	5	
subitem not at all important	○	○	○	◉	○	essential

Rensa markering

Does your paper address subitem X26-iii?

Copy and paste relevant sections from the manuscript (include quotes in quotation marks "like this" to indicate direct quotes from your manuscript), or elaborate on this item by providing additional information not in the ms, or briefly explain why the item is not applicable/relevant for your study

Participant privacy and data security were ensured through secure, password-protected systems and pseudonymization of study data. See "Written and verbal informed consent was obtained from each participant before participation in the study. Participant confidentiality was ensured by pseudonymisation as well as storing collected data and identifiers with restricted access. No compensation was provided for participation in this study."

Additionally, adverse events were monitored throughout the study period, and participants were instructed to contact the study team if concerns arose. However, this is not specified in the manuscript.

RESULTS

13a) For each group, the numbers of participants who were randomly assigned, received intended treatment, and were analysed for the primary outcome

NPT: The number of care providers or centers performing the intervention in each group and the number of patients treated by each care provider in each center

Does your paper address CONSORT subitem 13a? *

Copy and paste relevant sections from the manuscript (include quotes in quotation marks "like this" to indicate direct quotes from your manuscript), or elaborate on this item by providing additional information not in the ms, or briefly explain why the item is not applicable/relevant for your study

Yes, "A total of 654 patients were assessed for eligibility, 393 of whom did not meet the inclusion criteria, 164 declined participation and 37 did not respond. Therefore, 60 participants were randomized to the control or intervention group. However, after allocation into the groups one person was found to have been erroneously randomized. After exclusion of that person, a total of 59 participants were included in the analyses (intervention group n=29, 49%; control group n=30, 51%) (Figure 1). There were no withdrawals, major protocol violations, adverse events or deaths in either group."

Additionally, see the CONSORT flow diagram on page 9.

13b) For each group, losses and exclusions after randomisation, together with reasons

Does your paper address CONSORT subitem 13b? (NOTE: Preferably, this is shown in a CONSORT flow diagram) *

Copy and paste relevant sections from the manuscript (include quotes in quotation marks "like this" to indicate direct quotes from your manuscript), or elaborate on this item by providing additional information not in the ms, or briefly explain why the item is not applicable/relevant for your study

Yes, same as 13a. Additionally see figure 1, CONSORT flow diagram on page 9.

13b-i) Attrition diagram

Strongly recommended: An attrition diagram (e.g., proportion of participants still logging in or using the intervention/comparator in each group plotted over time, similar to a survival curve) or other figures or tables demonstrating usage/dose/engagement.

	1	2	3	4	5	
subitem not at all important	○	○	●	○	○	essential

Rensa markering

Does your paper address subitem 13b-i?

Copy and paste relevant sections from the manuscript or cite the figure number if applicable (include quotes in quotation marks "like this" to indicate direct quotes from your manuscript), or elaborate on this item by providing additional information not in the ms, or briefly explain why the item is not applicable/relevant for your study

Unfortunately, detailed engagement or usage data were not available; therefore, an attrition diagram was not included. However, the number of phone calls and health plan documentations is provided in the results "The median number of telephone calls in the intervention group during the six months was seven (ranging from two to ten telephone calls). All participants had a documented health plan on the platform, which was updated between one to seven times per participant (most often in connection with a telephone call)"

14a) Dates defining the periods of recruitment and follow-up

Does your paper address CONSORT subitem 14a? *

Copy and paste relevant sections from the manuscript (include quotes in quotation marks "like this" to indicate direct quotes from your manuscript), or elaborate on this item by providing additional information not in the ms, or briefly explain why the item is not applicable/relevant for your study

Yes, "Recruitment took place between August 2021 and June 2023" and "Participants answered questionnaires by post at baseline and at 3 and 6 months after inclusion (endpoint). "

14a-i) Indicate if critical "secular events" fell into the study period

Indicate if critical "secular events" fell into the study period, e.g., significant changes in Internet resources available or "changes in computer hardware or Internet delivery resources"

	1	2	3	4	5	
subitem not at all important	○	○	○	◉	○	essential

Rensa markering

Does your paper address subitem 14a-i?

Copy and paste relevant sections from the manuscript (include quotes in quotation marks "like this" to indicate direct quotes from your manuscript), or elaborate on this item by providing additional information not in the ms, or briefly explain why the item is not applicable/relevant for your study

No significant secular events (e.g., changes in Internet resources, hardware, or delivery infrastructure) occurred during the study period.

14b) Why the trial ended or was stopped (early)

Does your paper address CONSORT subitem 14b? *

Copy and paste relevant sections from the manuscript (include quotes in quotation marks "like this" to indicate direct quotes from your manuscript), or elaborate on this item by providing additional information not in the ms, or briefly explain why the item is not applicable/relevant for your study

Yes, "Because the inclusion rate was slower than expected, the research group deemed it infeasible to continue recruitment until desired statistical power was achieved. Thus, inclusion was terminated at 22 months, in June 2023."

15) A table showing baseline demographic and clinical characteristics for each group

NPT: When applicable, a description of care providers (case volume, qualification, expertise, etc.) and centers (volume) in each group

Does your paper address CONSORT subitem 15? *

Copy and paste relevant sections from the manuscript (include quotes in quotation marks "like this" to indicate direct quotes from your manuscript), or elaborate on this item by providing additional information not in the ms, or briefly explain why the item is not applicable/relevant for your study

Yes, a table showing baseline demographic and clinical characteristics is provided on page 10 in the manuscript.

15-i) Report demographics associated with digital divide issues

In ehealth trials it is particularly important to report demographics associated with digital divide issues, such as age, education, gender, social-economic status, computer/Internet/ehealth literacy of the participants, if known.

	1	2	3	4	5	
subitem not at all important	○	○	○	◉	○	essential

Rensa markering

Does your paper address subitem 15-i? *

Copy and paste relevant sections from the manuscript (include quotes in quotation marks "like this" to indicate direct quotes from your manuscript), or elaborate on this item by providing additional information not in the ms, or briefly explain why the item is not applicable/relevant for your study

Yes, partly. Some of these items are addressed in the table showing baseline demographic and clinical characteristics provided on page 10 in the manuscript. However, not computer/Internet/ehealth literacy of the participants.

16) For each group, number of participants (denominator) included in each analysis and whether the analysis was by original assigned groups

16-i) Report multiple “denominators” and provide definitions

Report multiple “denominators” and provide definitions: Report N’s (and effect sizes) “across a range of study participation [and use] thresholds” [1], e.g., N exposed, N consented, N used more than x times, N used more than y weeks, N participants “used” the intervention/comparator at specific pre-defined time points of interest (in absolute and relative numbers per group). Always clearly define “use” of the intervention.

	1	2	3	4	5	
subitem not at all important	○	○	◉	○	○	essential

Rensa markering

Does your paper address subitem 16-i? *

Copy and paste relevant sections from the manuscript (include quotes in quotation marks "like this" to indicate direct quotes from your manuscript), or elaborate on this item by providing additional information not in the ms, or briefly explain why the item is not applicable/relevant for your study

Multiple denominators across different usage thresholds were not reported. Outcomes were analyzed based on randomized group assignment.

16-ii) Primary analysis should be intent-to-treat

Primary analysis should be intent-to-treat, secondary analyses could include comparing only “users”, with the appropriate caveats that this is no longer a randomized sample (see 18-i).

	1	2	3	4	5	
subitem not at all important	○	○	○	◉	○	essential

Rensa markering

Does your paper address subitem 16-ii?

Copy and paste relevant sections from the manuscript (include quotes in quotation marks "like this" to indicate direct quotes from your manuscript), or elaborate on this item by providing additional information not in the ms, or briefly explain why the item is not applicable/relevant for your study

Yes, primary analysis is intent-to-treat (ITT) see manuscript "As there were no major protocol violations, only intention-to-treat (ITT) analysis was performed." Also the statistical analysis plan provided in Multimedia Appendix 3.

17a) For each primary and secondary outcome, results for each group, and the estimated effect size and its precision (such as 95% confidence interval)

Does your paper address CONSORT subitem 17a? *

Copy and paste relevant sections from the manuscript (include quotes in quotation marks "like this" to indicate direct quotes from your manuscript), or elaborate on this item by providing additional information not in the ms, or briefly explain why the item is not applicable/relevant for your study

Yes, see result table 2 on page 12 and result table 3 on page 13.

17a-i) Presentation of process outcomes such as metrics of use and intensity of use

In addition to primary/secondary (clinical) outcomes, the presentation of process outcomes such as metrics of use and intensity of use (dose, exposure) and their operational definitions is critical. This does not only refer to metrics of attrition (13-b) (often a binary variable), but also to more continuous exposure metrics such as “average session length”. These must be accompanied by a technical description how a metric like a “session” is defined (e.g., timeout after idle time) [1] (report under item 6a).

	1	2	3	4	5	
subitem not at all important	○	○	◉	○	○	essential

Rensa markering

Does your paper address subitem 17a-i?

Copy and paste relevant sections from the manuscript (include quotes in quotation marks "like this" to indicate direct quotes from your manuscript), or elaborate on this item by providing additional information not in the ms, or briefly explain why the item is not applicable/relevant for your study

Detailed usage or exposure metrics were not available; therefore, process outcomes such as intensity of use were not reported. However, the number of phone calls and health plan documentations on the eHealth platform is provided in the results "The median number of telephone calls in the intervention group during the six months was seven (ranging from two to ten telephone calls). All participants had a documented health plan on the platform, which was updated between one to seven times per participant (most often in connection with a telephone call)"

17b) For binary outcomes, presentation of both absolute and relative effect sizes is recommended

Does your paper address CONSORT subitem 17b? *

Copy and paste relevant sections from the manuscript (include quotes in quotation marks "like this" to indicate direct quotes from your manuscript), or elaborate on this item by providing additional information not in the ms, or briefly explain why the item is not applicable/relevant for your study

Not applicable; outcomes were not binary but were measured on a three-category ordered scale.

18) Results of any other analyses performed, including subgroup analyses and adjusted analyses, distinguishing pre-specified from exploratory

Does your paper address CONSORT subitem 18? *

Copy and paste relevant sections from the manuscript (include quotes in quotation marks "like this" to indicate direct quotes from your manuscript), or elaborate on this item by providing additional information not in the ms, or briefly explain why the item is not applicable/relevant for your study

Sensitivity analyses were done, with non-imputed data. See manuscript "A significant difference in composite score was also present in the non-imputed analysis (P=.035) (Table S3 in Multimedia Appendix 5)" and "a significance also found in the non-imputed analysis (Table S4 in Multimedia Appendix 5)"

18-i) Subgroup analysis of comparing only users

A subgroup analysis of comparing only users is not uncommon in ehealth trials, but if done, it must be stressed that this is a self-selected sample and no longer an unbiased sample from a randomized trial (see 16-iii).

	1	2	3	4	5	
subitem not at all important	○	◉	○	○	○	essential

Rensa markering

Does your paper address subitem 18-i?

Copy and paste relevant sections from the manuscript (include quotes in quotation marks "like this" to indicate direct quotes from your manuscript), or elaborate on this item by providing additional information not in the ms, or briefly explain why the item is not applicable/relevant for your study

Ditt svar

19) All important harms or unintended effects in each group
(for specific guidance see CONSORT for harms)

Does your paper address CONSORT subitem 19? *

Copy and paste relevant sections from the manuscript (include quotes in quotation marks "like this" to indicate direct quotes from your manuscript), or elaborate on this item by providing additional information not in the ms, or briefly explain why the item is not applicable/relevant for your study

Yes, " There were no withdrawals, major protocol violations, adverse events or deaths in either group"

19-i) Include privacy breaches, technical problems

Include privacy breaches, technical problems. This does not only include physical “harm” to participants, but also incidents such as perceived or real privacy breaches [1], technical problems, and other unexpected/unintended incidents. “Unintended effects” also includes unintended positive effects [2].

	1	2	3	4	5	
subitem not at all important	○	○	○	◉	○	essential

Rensa markering

Does your paper address subitem 19-i?

Copy and paste relevant sections from the manuscript (include quotes in quotation marks "like this" to indicate direct quotes from your manuscript), or elaborate on this item by providing additional information not in the ms, or briefly explain why the item is not applicable/relevant for your study

No unintended effects, privacy breaches or other unexpected incidents were reported during the study.

19-ii) Include qualitative feedback from participants or observations from staff/researchers

Include qualitative feedback from participants or observations from staff/researchers, if available, on strengths and shortcomings of the application, especially if they point to unintended/unexpected effects or uses. This includes (if available) reasons for why people did or did not use the application as intended by the developers.

	1	2	3	4	5	
subitem not at all important	○	○	○	◉	○	essential

Rensa markering

Does your paper address subitem 19-ii?

Copy and paste relevant sections from the manuscript (include quotes in quotation marks "like this" to indicate direct quotes from your manuscript), or elaborate on this item by providing additional information not in the ms, or briefly explain why the item is not applicable/relevant for your study

No, qualitative feedback from participants will be followed up on in a later process evaluation study.

DISCUSSION

22) Interpretation consistent with results, balancing benefits and harms, and considering other relevant evidence

NPT: In addition, take into account the choice of the comparator, lack of or partial blinding, and unequal expertise of care providers or centers in each group

22-i) Restate study questions and summarize the answers suggested by the data, starting with primary outcomes and process outcomes (use)

Restate study questions and summarize the answers suggested by the data, starting with primary outcomes and process outcomes (use).

	1	2	3	4	5	
subitem not at all important	○	○	○	●	○	essential

Rensa markering

Does your paper address subitem 22-i? *

Copy and paste relevant sections from the manuscript (include quotes in quotation marks "like this" to indicate direct quotes from your manuscript), or elaborate on this item by providing additional information not in the ms, or briefly explain why the item is not applicable/relevant for your study

Yes "To our knowledge, this is the first trial investigating the efficacy of a person-centred intervention carried out remotely, consisting of a combined telephone support and eHealth platform, for persons on sick leave due to chronic pain. Our results showed a statistically significant difference in the composite score for GSE and sick leave, favouring the intervention group receiving PCC. The findings suggest that PCC may have a protective role, as only three participants in the intervention group had deteriorated by the 6-month follow-up, compared with eleven in the control group."

22-ii) Highlight unanswered new questions, suggest future research

Highlight unanswered new questions, suggest future research.

	1	2	3	4	5	
subitem not at all important	○	○	○	○	●	essential

Rensa markering

Does your paper address subitem 22-ii?

Copy and paste relevant sections from the manuscript (include quotes in quotation marks "like this" to indicate direct quotes from your manuscript), or elaborate on this item by providing additional information not in the ms, or briefly explain why the item is not applicable/relevant for your study

Yes, "Future research may benefit from further exploration of differences between patient groups, implementation processes and mechanisms, and assess the long-term outcomes of the EAPER-P intervention."

20) Trial limitations, addressing sources of potential bias, imprecision, and, if relevant, multiplicity of analyses

20-i) Typical limitations in ehealth trials

Typical limitations in ehealth trials: Participants in ehealth trials are rarely blinded. Ehealth trials often look at a multiplicity of outcomes, increasing risk for a Type I error. Discuss biases due to non-use of the intervention/usability issues, biases through informed consent procedures, unexpected events.

	1	2	3	4	5	
subitem not at all important	○	○	○	◉	○	essential

Rensa markering

Does your paper address subitem 20-i? *

Copy and paste relevant sections from the manuscript (include quotes in quotation marks "like this" to indicate direct quotes from your manuscript), or elaborate on this item by providing additional information not in the ms, or briefly explain why the item is not applicable/relevant for your study

Partly, typical trial limitations, including recruitment, data collection and statistical managment is discussed in the Discussion section. However, non-use attrition has not been specifically mentioned due to lack of availability of that type of data.

21) Generalisability (external validity, applicability) of the trial findings

NPT: External validity of the trial findings according to the intervention, comparators, patients, and care providers or centers involved in the trial

21-i) Generalizability to other populations

Generalizability to other populations: In particular, discuss generalizability to a general Internet population, outside of a RCT setting, and general patient population, including applicability of the study results for other organizations

	1	2	3	4	5	
subitem not at all important	○	○	○	○	●	essential

Rensa markering

Does your paper address subitem 21-i?

Copy and paste relevant sections from the manuscript (include quotes in quotation marks "like this" to indicate direct quotes from your manuscript), or elaborate on this item by providing additional information not in the ms, or briefly explain why the item is not applicable/relevant for your study

Yes, "One strength of the intervention includes that, a third of the study population were born outside of Sweden, which closely reflects the population living in the region (58). Having a diverse participant population is important as country of birth has been shown to affect the severity of clinical presentations of pain as well as affecting outcomes of pain rehabilitation (59). This could therefore enhance generalizability of the results."

"the intervention was evaluated in an efficacy trial and was therefore administered in a research context with ideal conditions, e.g. with HCPs dedicated solely to working on the study. This factor may impact the transferability of the results to a clinical setting. However, measuring efficacy instead of effectiveness often means that the internal validity is higher and that the efficacy can be estimated with less bias (55). In a clinical setting, interventions can also lead to spill-over effects, where outcomes extend beyond those intended, especially when HCPs are involved in multiple activities or in treating multiple groups, and move between units or share common social areas (56)."

21-ii) Discuss if there were elements in the RCT that would be different in a routine application setting

Discuss if there were elements in the RCT that would be different in a routine application setting (e.g., prompts/reminders, more human involvement, training sessions or other co-interventions) and what impact the omission of these elements could have on use, adoption, or outcomes if the intervention is applied outside of a RCT setting.

	1	2	3	4	5	
subitem not at all important	○	○	○	◉	○	essential

Rensa markering

Does your paper address subitem 21-ii?

Copy and paste relevant sections from the manuscript (include quotes in quotation marks "like this" to indicate direct quotes from your manuscript), or elaborate on this item by providing additional information not in the ms, or briefly explain why the item is not applicable/relevant for your study

Yes, partly discussed in the following segment "the intervention was evaluated in an efficacy trial and was therefore administered in a research context with ideal conditions, e.g. with HCPs dedicated solely to working on the study. This factor may impact the transferability of the results to a clinical setting. However, measuring efficacy instead of effectiveness often means that the internal validity is higher and that the efficacy can be estimated with less bias (55). In a clinical setting, interventions can also lead to spill-over effects, where outcomes extend beyond those intended, especially when HCPs are involved in multiple activities or in treating multiple groups, and move between units or share common social areas (56). "

OTHER INFORMATION

23) Registration number and name of trial registry

Does your paper address CONSORT subitem 23? *

Copy and paste relevant sections from the manuscript (include quotes in quotation marks "like this" to indicate direct quotes from your manuscript), or elaborate on this item by providing additional information not in the ms, or briefly explain why the item is not applicable/relevant for your study

Yes, on page 2 "ClinicalTrials.gov identifier: NCT04706195, submitted 2021-01-09."

24) Where the full trial protocol can be accessed, if available

Does your paper address CONSORT subitem 24? *

Cite a Multimedia Appendix, other reference, or copy and paste relevant sections from the manuscript (include quotes in quotation marks "like this" to indicate direct quotes from your manuscript), or elaborate on this item by providing additional information not in the ms, or briefly explain why the item is not applicable/relevant for your study

There is no full trial protocol published, however trial registry is available as per above, additionally the Statistical Analysis Plan is submitted through Multimedia appendix 3.

25) Sources of funding and other support (such as supply of drugs), role of funders

Does your paper address CONSORT subitem 25? *

Copy and paste relevant sections from the manuscript (include quotes in quotation marks "like this" to indicate direct quotes from your manuscript), or elaborate on this item by providing additional information not in the ms, or briefly explain why the item is not applicable/relevant for your study

Yes, on page 17 "Funding: This work was supported by FORTE (grant No. 2019-00718) and the University of Gothenburg Centre for Person-Centred Care (GPCC) (grant number N/A)."

X27) Conflicts of Interest (not a CONSORT item)

X27-i) State the relation of the study team towards the system being evaluated

In addition to the usual declaration of interests (financial or otherwise), also state the relation of the study team towards the system being evaluated, i.e., state if the authors/evaluators are distinct from or identical with the developers/sponsors of the intervention.

	1	2	3	4	5	
subitem not at all important	○	○	○	○	◉	essential

Rensa markering

Does your paper address subitem X27-i?

Copy and paste relevant sections from the manuscript (include quotes in quotation marks "like this" to indicate direct quotes from your manuscript), or elaborate on this item by providing additional information not in the ms, or briefly explain why the item is not applicable/relevant for your study

Yes, "The eHealth platform was developed at the University of Gothenburg through collaboration among information technology (IT) developers, HCPs, researchers, and research partners, where the research partners were active in its development through a participatory process"

About the CONSORT EHEALTH checklist

As a result of using this checklist, did you make changes in your manuscript? *

- ○ yes, major changes
- ◉ yes, minor changes
- ○ no

What were the most important changes you made as a result of using this checklist?

Very minor, title, COI etc

How much time did you spend on going through the checklist INCLUDING making changes in your manuscript *

approximately half a workday/4 hours

As a result of using this checklist, do you think your manuscript has improved? *

- (x) yes
- ( ) no
- ( ) Övrigt:

Would you like to become involved in the CONSORT EHEALTH group?

This would involve for example becoming involved in participating in a workshop and writing an "Explanation and Elaboration" document

- ( ) yes
- (x) no
- ( ) Övrigt:

Rensa markering

Any other comments or questions on CONSORT EHEALTH

1. Some questions are very long/multifacetted, however does not allow long answers which is a limitation
2. Some items are repetitive or near identical, making the checklist very long

STOP - Save this form as PDF before you click submit

To generate a record that you filled in this form, we recommend to generate a PDF of this page (on a Mac, simply select "print" and then select "print as PDF") before you submit it.

When you submit your (revised) paper to JMIR, please upload the PDF as supplementary file.

Don't worry if some text in the textboxes is cut off, as we still have the complete information in our database. Thank you!

Final step: Click submit !

Click submit so we have your answers in our database!

Skicka

Rensa formuläret

Skicka aldrig lösenord med Google Formulär

Det här innehållet har varken skapats eller godkänts av Google. - Ägare av kontaktformulär - Användarvillkor - Integritetspolicy

Ser det här formuläret misstänkt ut? Rapport

Google Formulär
