# Supplementary material for: Efficacy of a Remote Person-Centered Intervention Using an eHealth Platform and Telephone Support for Persons With Chronic Pain: Randomized Controlled Trial
Source: JMIR Form Res. 2026 Aug 3;10:e91887. doi: 10.2196/91887 (PMC13432249; doi:10.2196/91887)
Supplement: Checklist 2 [file formative-v10-e91887-s005.pdf]

GRIPP2 short form

Section and topic	Item	Reported on page No
1: Aim	Report the aim of PPI in the study	9, 18
2: Methods	Provide a clear description of the methods used for PPI in the study	9, 18
3: Study results	Outcomes—Report the results of PPI in the study, including both positive and negative outcomes	18
4: Discussion and conclusions	Outcomes—Comment on the extent to which PPI influenced the study overall. Describe positive and negative effects	9, 18
5: Reflections/critical perspective	Comment critically on the study, reflecting on the things that went well and those that did not, so others can learn from this experience	18

PPI=patient and public involvement
